# Pearl Millet Genetic Traits Shape Rhizobacterial Diversity and Modulate Rhizosphere Aggregation

**DOI:** 10.3389/fpls.2017.01288

**Published:** 2017-07-27

**Authors:** Papa M. S. Ndour, Mariama Gueye, Mohamed Barakat, Philippe Ortet, Marie Bertrand-Huleux, Anne-Laure Pablo, Damien Dezette, Lydie Chapuis-Lardy, Komi Assigbetsé, Ndjido Ardo Kane, Yves Vigouroux, Wafa Achouak, Ibrahima Ndoye, Thierry Heulin, Laurent Cournac

**Affiliations:** ^1^IRD, UMR Eco&Sols, LMI IESOL, Centre de recherche ISRA-IRD Dakar, Sénégal; ^2^Département de Biologie Végétale, Université Cheikh Anta Diop Dakar, Sénégal; ^3^Aix Marseille Université, CEA, CNRS, UMR7265, LEMIRE, Laboratoire d'Ecologie Microbienne de la Rhizosphère et Environnement extrêmes, ECCOREV FR3098 F-13108 St Paul Les Durance, France; ^4^Eco&Sols, Cirad, INRA, IRD, Montpellier SupAgro, Université de Montpellier Montpellier, France; ^5^Laboratoire National de Recherches sur les Productions Végétales, Institut Sénégalais de Recherches Agricoles, Centre de Recherche de Bel Air Dakar, Senegal; ^6^IRD UMR DIADE Montpellier, France

**Keywords:** pearl millet, rhizosphere, soil aggregation, 16S rDNA sequencing, metabarcoding, EPS producing bacteria, rhizosheath

## Abstract

Root exudation contributes to soil carbon allocation and also to microbial C and energy supply, which subsequently impacts soil aggregation around roots. Biologically-driven soil structural formation is an important driver of soil fertility. Plant genetic determinants of exudation and more generally of factors promoting rhizosphere soil aggregation are largely unknown. Here, we characterized rhizosphere aggregation in a panel of 86 pearl millet inbred lines using a ratio of root-adhering soil dry mass per root tissue dry mass (RAS/RT). This ratio showed significant variations between lines, with a roughly 2-fold amplitude between lowest and highest average values. For 9 lines with contrasting aggregation properties, we then compared the bacterial diversity and composition in root-adhering soil. Bacterial α-diversity metrics increased with the “RAS/RT ratio.” Regarding taxonomic composition, the *Rhizobiales* were stimulated in lines showing high aggregation level whereas *Bacillales* were more abundant in lines with low ratio. 184 strains of cultivable exopolysaccharides-producing bacteria have been isolated from the rhizosphere of some lines, including members from *Rhizobiales* and *Bacillales*. However, at this stage, we could not find a correlation between abundance of EPS-producing species in bacterial communities and the ratio RAS/RT. These results illustrated the impact of cereals genetic trait variation on soil physical properties and microbial diversity. This opens the possibility of considering plant breeding to help management of soil carbon content and physical characteristics through carbon rhizodeposition in soil.

## Introduction

The last decades in Sahelian regions led to shorter rainy seasons. As a response, farmers in Niger have selected for short-cycle millet [*Pennisetum glaucum* (L) R.Br] varieties (Vigouroux et al., 2011a). This adaptation has been based on the available genetic diversity of the species, including the genetic variability for the duration of its growth cycle (Vigouroux et al., 2011b). If above ground phenotypic diversity has been consequently largely documented, phenotypic diversity studies on belowground characters are scarce. However, roots and soils are key components for adaptation of crops since they impact the availability of water and minerals. The properties of structural units of soil (i.e., size and stability of soil aggregates) determine overall soil physical and mechanical properties such as retention and movement of water, aeration, and temperature that have direct impacts on plant growth (Dickson et al., 1991). This aggregation is strongly influenced by carbon content of soil (Haynes and Swift, 1990). Indeed, aggregate stability is a key factor for the soil physical fertility, it also affects soil organic matter dynamics (Six et al., 2000; Abiven et al., 2009). Improvement of such properties is commonly envisioned by the management of organic fertilization or tillage practices. In the Sahelian cropping systems where aerial plant residues are often exported for feeding livestock, root carbon deposition may serve as an important source of soil fresh organic matter. Root exudates may represent up to 17% of plant photo-assimilates (Nguyen, 2003). Carbon from root exudation is a preferred source of C and energy for soil microbiota, particularly for rhizobacteria. Some of these rhizobacteria synthesize biopolymers such as exopolysaccharides (EPS) that also contribute to the stabilization of soil aggregates in the rhizosphere by cementing particles together (Bezzate et al., 2000).

We know that EPS-producing bacteria inoculation can impact rhizosphere soil structure (Amellal et al., 1998; Alami et al., 2000; Berge et al., 2009). After inoculation of wheat with a EPS-producing *Pantoea agglomerans* strain, the root-adhering soil per unit of root mass increased by 50% (Amellal et al., 1998). In addition, the macro-porosity of root-adhering soil was improved (pores diameter between 10 and 30 μm). The inoculation of sunflower with a EPS-producing strain of *Rhizobium alamii* increased the root-adhering soil per root mass ratio (up to 100%), soil macropore volume (pores diameter between 10 and 60 μm), as well as root and shoot dry mass under normal and water stress condition (Alami et al., 2000; Berge et al., 2009). These bacterial strains essentially rely on plant-based carbon sources in soil. Therefore, it can be hypothesized that plant inter- and intra-specific diversity in rhizodeposition may also yield differential impacts on the diversity and activity of soil microbiota.

Several studies also found intra-specific plant genetic traits impact on diversity of rhizomicrobial community: in maize (Peiffer et al., 2013), in *Arabidopsis thaliana* (Micallef et al., 2009) and in *Medicago truncatula* (Zancarini et al., 2012). However, the consequences of such variety-dependent variations in bacterial community on soil properties is not well established. On an other hand, genetic variations have been found on the amount of soil closely adhering to root (also called rhizosheath) in some cereals (Delhaize et al., 2012; George et al., 2014). In wheat, intra-specific variation of these structures in acid soil was significantly correlated with length of root hairs and Al tolerance (Delhaize et al., 2012). In barley, rhizosheath formation was variable between 144 varieties in both greenhouse and field conditions and was associated with a genetic locus (QTL) on chromosome 2H (George et al., 2014). Moreover, this rhizosheath size variation had an impact on plant growth under water and phosphate deficiency stress (George et al., 2014).

Therefore, if plant intra-specific diversity exists, cereal varieties promoting soil aggregation *via* an optimized root carbon exudation could be selected. Indeed, these traits are expected to contribute to maintaining soil structure/fertility and to sequestering carbon in soil. Such selection targets could therefore be considered toward global concerns such as agriculture sustainability or mitigation of atmospheric CO_2_ increase. Note however that it is still unclear how much of exudate carbon remains in soil after growing season and would therefore significantly contribute to C sequestration. It might be an interesting challenge to check how EPS-organic matter production and protection from microbial degradation in aggregate structures could contribute to progressively and slightly increase soil carbon content.

Our study aims to determine whether there is an intra-specific variability of millet rhizodeposition, and more generally of phenomena leading to root-driven soil aggregation and microbiological diversity. Direct assessment of root exudation *in natura* is not an easy task and can hardly be envisioned for high throughput phenotyping. Based on the links between soil adherence to roots, aggregation, microbial activities and root exudation as detailed above, we decided to assess the diversity of the ratio between mass of root-adhering soil and mass of root tissue (Amellal et al., 1998) as a phenotyping proxy for these phenomena. We further assessed how this ratio is associated with the diversity of bacterial community in the rhizosphere.

## Materials and methods

We performed a wide screening of 86 pearl millet inbreed lines previously described (Saïdou et al., 2009). Our phenotyping is based on the ratio between dry mass of root-adhering soil and dry mass of root tissue (RAS/RT) (Amellal et al., 1998; Alami et al., 2000; Bezzate et al., 2000). After this screening, we subsequently validated the phenotype variation of the most extreme lines in two additional experiments with 16 and 9 of the most contrasted pearl millet inbred lines. For the last experiment with 9 different lines, we then carried out an analysis of the diversity of rhizosphere microbiota, including a focus on EPS-producing bacterial populations.

### Soil

The sandy soil we used in this experiment was sampled in surface horizon (0–20 cm) at ISRA CNRA station of Bambey (14°42′38.7″N and 16°28′47.2″W), sieved at 4 mm and homogenized. This sampling site is located in the groundnut basin of Senegal where pearl millet is the first cultivated cereal. The soil type, common in the area, is arenosol according to the world reference base for soil resources (FAO, 2006). The soil texture characteristics were 94.5% sand, 3% silt, and 2.7% clay and pH was 7.7 (measured at 1:1 soil water ratio). Total carbon and nitrogen concentrations were 0.69 and 0.04% respectively.

### Inbred lines

We tested 86 pearl millet [*P. glaucum* (L) R.Br.] inbred lines originating from Niger, Togo, Senegal, Zimbabwe, and Cameroon which were used in a previous genetic association study (Saïdou et al., 2009). These lines were obtained from the International Crop Research Institute for Semi-Arid Tropics (ICRISAT), the Centre de Coopération Internationale en Recherche Agronomique pour le Développement (CIRAD) and the University of Orsay (Paris XI). The greenhouse phenotyping experiment was carried out in 7 successive blocks of 12 to 14 lines each, with five repeats per line. We indicated the block in which each line was phenotyped in supplementary data (Table S1). To detect variability among phenotyping series, two pearl millet lines (L82 and L114) were repeatedly phenotyped in three different blocks as controls.

### Seeding and plant growth

Soil was filled in bottomless “WM” shaped pots that were installed in plastic crates at the bottom of which we spread an anti-mosquito canvas to prevent soil loss. These pots are constituted by two easily detachable nested parts, and are particularly convenient for root phenotyping purposes. Moreover, their angular shape prevents root spiraling. Each pot contained 1.5 kg of soil and five replicates were sown for each line with four seeds per pot. Experiments were conducted under natural light in a greenhouse. After 1 week of growth, thinning was performed to have one plant per pot. The soil moisture was daily adjusted at water-holding capacity (WHC) by watering each pot with 15–20 mL. Plant watering was stopped 24 h before harvesting to facilitate the separation of root-adhering soil (RAS) from bulk soil.

### Harvest and phenotype comparison

After 28 days of growth, plants were harvested at laboratory and treated as follows: first we gently removed the two parts of the pot to better keep the roots with their adhering soil. Then, plants were fixed (at the crown level) on an electric agitator (S50, CAT, Staufen, Germany) and shaken with a constant speed of 1,100 rpm during 1 min to separate the roots from non-adhering soil in a repeatable way (Figure S1). The root-adhering soil was recovered by washing the roots in sterile distilled water using aluminum weighing dishes. Roots and shoots were collected and incubated at 65°C for 3 days to measure their dry masses (root dry mass is also abbreviated as RT for “root tissue”). A sample of bulk soil was also systematically taken to measure soil moisture at harvest. The RAS was dried at 105°C for 3 days and weighted allowing the calculation of RAS/RT ratio.

### Comparison of contrasted inbred lines

After the screening of the 86 pearl millet inbred lines, 16 lines with high or low RAS/RT ratio values were selected. For lines selection we also took into account plant development, germplasm availability, and phenotype homogeneity. They were sown in the same conditions as for the screening experiment in order to confirm their contrasted phenotype within a single cultivation batch. The growth and harvest protocol was the same as that described previously. For analyses of bacterial community, we further selected 9 lines among the 16 as statistically separated: 3 lines with low RAS/RT ratio (<15 g/g) and 6 lines with high RAS/RT ratio (>20 g/g). These 9 lines were phenotyped in another batch with 10 replicates per line.

### Rhizosphere bacterial community analysis

The 9 selected millet lines were sown in another experiment in the same soil and always the same cultivation protocol. We included in this experiment four pots without plant that were considered as a control. For each line, four plants were sown and after 28 days of growth, we performed the harvest. For each plant we sampled and homogenized separately the totality of root-adhering soil and the bulk soil in the pot (soil not adhering to roots). We also took four independent control soil samples, one from each of the unplanted pots kept in the same conditions as cultivated ones. The total DNA was extracted from 0.25 g of root-adhering soil and control soil samples using FastDNA spin Kit for soil (Q-Biogene, Illkirch, France), according to the supplier's instructions. DNA amounts in extracts were quantified by UV absorbance using a Nanodrop 2000 spectrophotometer (Thermo Fischer Scientific, Waltham, Massachusetts, USA).

### Bacterial density analysis

Bacterial density was estimated by real-time quantitative polymerase chain reaction (q-PCR) of ribosomal DNA. This approach is now considered as a valuable, accurate and culture-independent method (Smith and Osborn, 2009). Amplification was carried out with a CFX96 Touch™ Real-Time PCR Detection System (Biorad, Hercules, CA, USA) using SYBR Green as detection system. For amplification, we performed PCR in 10 μL of reaction mixture composed by 0.5 μM for each of the primers 341 F (5′ CCT ACG GGA GGC AGC AG 3′) and 515R (5′ ATT ACC GCG GCT GCT GGC A 3′), 5 μL of SsoAdvanced SYBR Green mix (Biorad, USA), 2.5 ng of template DNA and DNAse-RNAse free water. The cycling program included: 10 min of incubation at 98°C, followed by 39 cycles of 98°C for 5 s and 60°C for 30 s. Amplification specificity was studied using melting curve analysis of the PCR products performed by ramping the temperature from 55 to 95°C (+0.5°C every 5 s). Standard tubes containing DNA copies numbers ranging from 10^3^ to 10^8^ were used for calibration.

### Bacterial diversity analysis

Sequencing of gene encoding 16S rRNA from DNA extracts was performed at MR DNA (Shallowater, TX, USA) with an Ion Torrent PGM system for sequencing (Life Technologies Corp., Thermo Fischer Scientific, Massachusetts, USA). Samples were barcoded and the V4 variable region of 16S rRNA gene was targeted with 515F/806R primers set (GTGCCAGCMGCCGCGGTAA–GGACTACHVGGGTWTCTAAT) (Caporaso et al., 2011). PCR amplification was carried out using the HotStar Taq plus Master Mix Kit (Qiagen, Germantown, MD, USA). The conditions were as follows: 94°C for 3 min, 28 cycles of 94°C for 30 s, 53°C for 40 s, and 72°C for 1 min. After this, a final elongation step of 72°C for 5 min was performed. Sequences data analyses were conducted using QIIME software version 1.8 with standard parameters (Caporaso et al., 2010b). Overall sequences with quality score average <25, short sequences (<200 bp) and sequences containing mismatches as well as homopolymers were removed from the analysis. We conducted a denoising of sequences and a chimera check and filtered mitochondria and chloroplasts sequences using an in-house PHP script (from CEA Cadarache, France). Thus, the multiplex reads were assigned to corresponding sample according to their barcode, and using the Usearch scripts we carried out Operational Taxonomic Unit (OTU) picking by *de novo* checking method (Edgar, 2010). Representative sequences of the OTUs were aligned using the PyNAST algorithm with a minimum percent of 80% (Caporaso et al., 2010a). These OTUs were taxonomically classified using RDP method and Greengenes database (DeSantis et al., 2006). The phylogenetic position of bacteria in the different rhizosphere lines was characterized using taxa summary QIIME script until genus level (L6). To compare bacterial OTU richness and diversity index in the rhizosphere between inbred lines with these different numbers of sequences, we performed a rarefaction analysis i.e., a random picking of an equal sequence number in each sample (40,870 reads per sample) by using the open-source bioinformatics pipeline QIIME (Caporaso et al., 2010b). α-diversity parameters [number of observed OTUs, qualitative Chao1 index, Faith's Phylogenetic Diversity (PD_whole), and Shannon index] were then calculated on 40,870 randomly picked sequences for each sample. Sequence data are available in NCBI SRA bank under accession number *SRR5482172*, experiment *SRX2708874">SRX2708874*.

### Cultivable bacterial analysis

Experimentally the root-adhering soil per root mass ratio was reported to be increased after EPS-producing rhizobacterial inoculation (Kaci et al., 2005). Therefore, we aimed to cultivate and characterize EPS-producing rhizobacterial populations from the selected “contrasted” millet lines. Putative EPS-producing strains can be identified using the mucoid phenotype of the colonies on C-enriched solid medium (Hebbar et al., 1992). Doing so, we estimated as well the abundance of cultivable bacteria in rhizosphere soil. For each line, we sampled four root-adhering soil fractions and four bulk soil samples. For each sample we dispersed 1 g of RAS or bulk soil in 3 mL of sterile distilled water and performed serial dilutions until 10^−4^ with KCl solution at 0.85%. Plating on Petri dishes was performed with 10 μL of the two highest dilutions for each sample (10^−3^ and 10^−4^) on three different media: Tryptic Soy Broth medium 10-fold diluted + agar 15 g.L^−1^ (TSA/10), TSA/10 + glucose (20 g.L^−1^) and TSA/10 + sucrose (20 g.L^−1^). Supplementation of TSA/10 with high concentration of glucose or sucrose was chosen for the selection of EPS-producing bacterial strains (Hebbar et al., 1992). All these media contained cycloheximide (70 mg.L^−1^) serving as antifungal agent. We finally counted the number of cultivable bacteria (CFU, Colony-Forming Units) in Petri dishes spread with the 10^−4^ soil dilution after 48 h of growth. On the same plates we selected putative EPS-producing bacterial strains based on the morphological aspect of the colonies: mucoid spreading or thick capsular formation on C-enriched medium. We pricked out and purified these EPS-producing strains on TSA/10 and then subcultured them in diluted Tryptic Soy Broth medium (TSB/10) during 24–48 h before storage in glycerol at −80°C. For identification of bacterial strains, we carried out PCR directly on colonies to amplify the gene encoding 16S rRNA using Fd1/Rd1 primers (Weisburg et al., 1991), and sequenced the amplicons at Cogenics, Inc. (Morrisville NC, USA). Then sequences were aligned and 48 consensus sequences were defined and used for an NCBI blast in RDP II database to perform taxonomic assignment.

To determine the abundance of these EPS-producing bacterial species (or closely related species) in the rhizosphere of the 9 pearl millet inbred lines, we used the BLAST software (version 2.3.0+) from NCBI and we performed a blast data search with the OTU sequences derived from PGM sequencing. Cultivable bacterial species identified with the sequence of the gene encoding 16S rRNA (sequence length ranging from 707 to 1,423 bp) were targeted in this blast. An identity percentage of 97% at least was fixed to validate match between a cultivable bacterial species sequence and an OTU sequence. Sequences of strains are available on NCBI SRA under accession numbers *KY880842* to *KY880971*.

### Statistical analyses

We used XLSTAT (Addinsoft SARL, Paris, France) for statistical analysis. The normality of different variables was tested using Shapiro-Wilk test and the homoscedasticity by Levene test. For RAS/RT ratio the data were square root transformed to improve normality before ANOVA modeling. Analysis of Variance (ANOVA) and Tukey pairwise comparison test (95%) were performed for RAS/RT ratios of pearl millet lines. Pearson correlation test (95%) was used to assess the relations between RAS/RT ratios, root mass, shoot mass and soil moisture at harvest. For the 9 selected lines, we compared the number of 16S rDNA copies per gram of dried soil using ANOVA and a Fisher pairwise test (95%; *n* = 4). Kruskal-Wallis tests were performed to compare rhizosphere α-diversity metrics to those of the control soil and to compare *phylum* and *order* relative abundances between different treatments. Weighted and Unweighted UniFrac distance matrixes were calculated from the OTU table and used to perform Principal Coordinate Analysis (PCoA) and to compare β-diversity of the different treatments. The significance of different clusters separation in the PCoA was tested using PERMANOVA (999 permutations) of weighted and unweighted UniFrac distance matrixes. We carried out Principal Component Analysis (PCA) to study the relation between soil aggregation (RAS/RT), shoot mass, root mass and the presence of the bacterial orders we found in the RAS by the 16S PGM sequencing. To do this we used for each plant line the mean frequencies of the 26 most abundant bacterial orders in rhizosphere and averages of plant parameters (RAS/RT ratio, shoot, and root dry mass) from the third phenotyping experiment.

## Results

### Significant changes between pearl millet inbred lines for rhizosphere soil aggregation (RAS/RT ratio)

**Figure 1:**
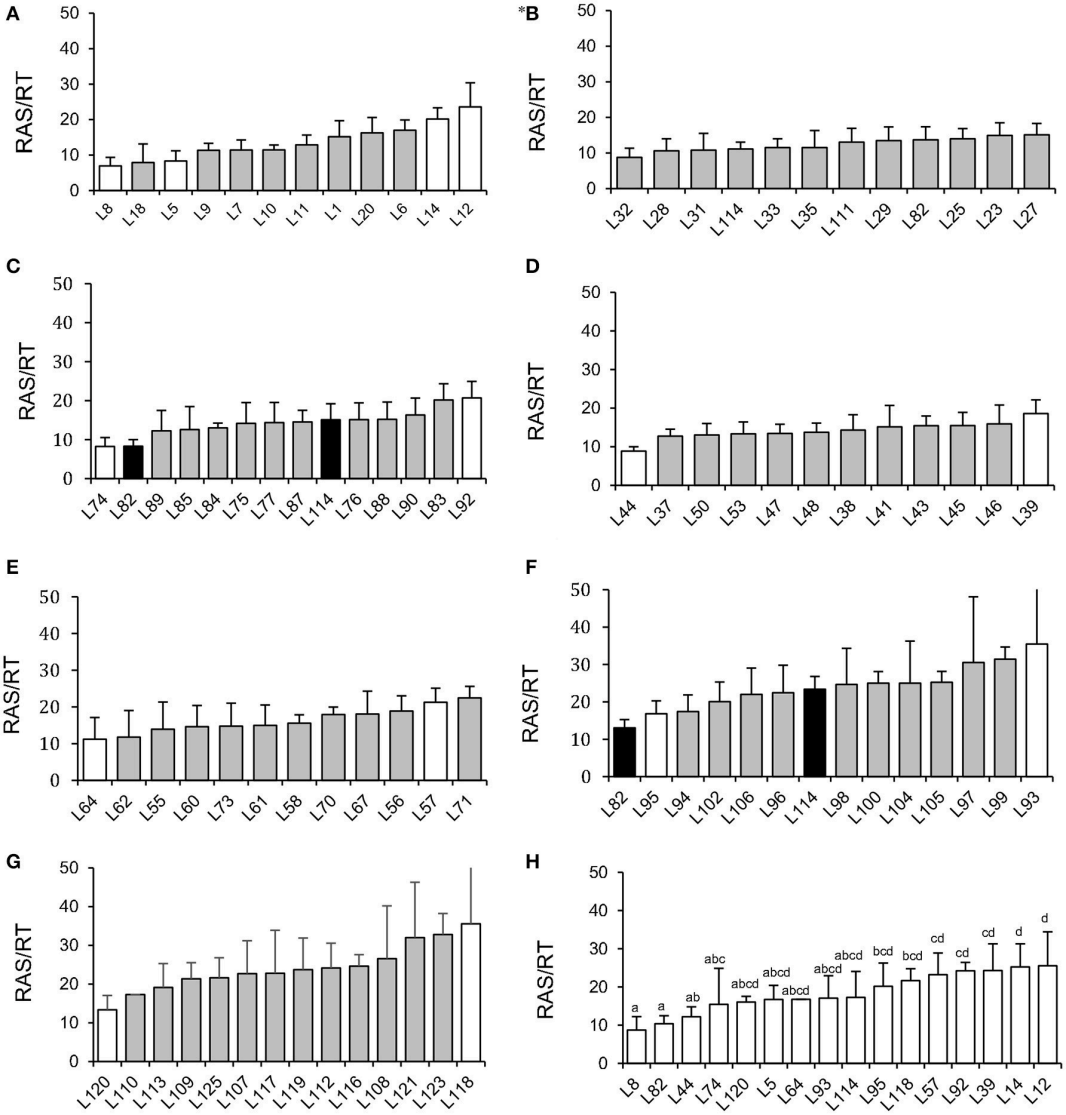
Screening of root-adhering soil (g dm) per root tissue mass (g dm) (RAS/RT ratio) of 86 pearl millet inbred lines **(A–G)**: In each graph we arranged lines' (RAS/RT) phenotype in order from the smallest to the largest values recorded in a given block. ^*^(B) the *p*-value of the ANOVA testing for the millet line effect was non-significant for this block. The white histograms indicate the 16 selected lines phenotyped in the second experiment. The black histograms indicate the 2 lines used as control for inter-block variation check. Graph **(H)** represents phenotype verification's results for 16 selected lines from the screening experiment. The bar plots represent means of five repeats and error bars represent standard deviations, letters represent Fisher LSD groups.

The screening experiment showed significant effect of millet line (*p* < 0.05, tested by ANOVA in each block, Table S1) on recorded RAS/RT in most of the culture blocks (Figures 1A–G), except for block 2 (Figure 1B). Average value per line ranged from 6.9 g/g (L8) to 35.6 g/g (L118), with therefore a 5-fold factor between extreme values (Figure 1). As soil moisture is known to potentially affect soil aggregation and adherence properties, we also checked correlation between RAS/RT ratio and soil moisture at harvest. No significant correlation was found (*R* = 0.041 *p* > 0.3), indicating that our control of soil moisture was sufficient to prevent this parameter for having a major influence on aggregation phenotyping.

Since the screening was performed in different blocks, two pearl millet lines (L82 and L114) that were present in block 2 were repeatedly introduced in blocks 5 and 6 to evaluate the inter-block variability. This assay pointed out a *ca*. 2-fold variability of average RAS/RT ratio among these blocks, from 15 to 23 g/g (L114) and from 8 to 13 g/g (L82), but the ranking was conserved between the two lines. We consequently undertook two new experiments to confirm the phenotypes of contrasted lines and retain 9 of them for microbial analysis.

### Confirmation of variation in RAS/RT ratio

**Figure 2:**
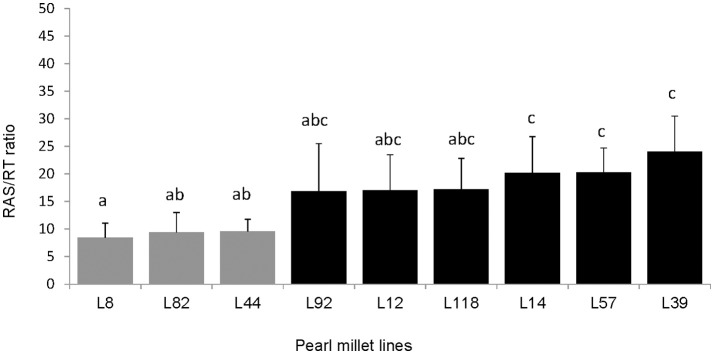
Comparison of root-adhering soil (g dm) per root tissue mass (g dm) (RAS/RT ratio) of the 9 pearl millet inbred lines (*n* = 10 per line) which were selected from the 16 lines for their low (gray) vs. high (black) ratio. The bars on the histogram represent the standard deviations. Different letters indicate significant differences with Tukey's Honest Significant Difference test (*p* < 0.05).

To continue our study with a smaller number of pearl millet lines, we selected firstly 16 contrasted lines among the 86 lines screened in the previous experiment. These lines were selected from the blocks where statistically significant effects were found, and chosen as exhibiting average RAS/RT values close to the lowest or highest value observed within the block. Other parameters of choice were: availability of germplasm, phenological homogeneity, germination success. We conducted a second phenotyping experiment on these 16 lines in a single batch. We observed significant difference of the ratio RAS/RT between lines and a global coherence in phenotype between this batch and previous experiment. For instance, L8, L82, and L44 which were selected from the lines expressing relatively low RAS/RT ratios in blocks during the whole set phenotyping, also had lower RAS/RT ratio compared to the others lines in this batch (Figure 1H). For the third experiment (same conditions than during screening, but now 10 repeats per line in order to enhance strength of the ANOVA analysis) we selected 9 lines from the 16, taking two groups which appeared statistically separated in the 16-lines experiments, i.e., L8, L44, and L82 with low RAS/RT values, vs. L12, L14, L39, L57, L92, and L118 with high RAS/RT values. The separation between these groups appeared consistent in this third experiment (Figure 2). Again the ratio RAS/RT was not correlated with soil moisture (Table 1). The RAS was positively correlated with root and shoot biomass: plants with larger root development mechanically carry more root-adhering soil. On the other hand, RAS/RT ratio was negatively correlated with shoot biomass, which might be in line with an increased C sink in root exudation. When pooled together, the two groups of lines “low RAS/RT ratio” (L8, L82, and L44) and “high RAS/RT ratio” (L92, L12, L118, L14, L57, and L39) were clearly separated in terms of average RAS/RT ratio, the difference being highly significant (respectively 9.15 < 19.35 g/g; Student *t*-test *p* < 0.0001).

**Table 1:** Correlation matrix between parameters recorded during phenotyping of 9 pearl millet inbred lines (3rd experiment).

	**Shoot dm**	**Root (RT) dm**	**RAS dm**	**RAS/RT ratio**	**Moisture**
Shoot dm	1	**0.655**	**0.229**	−**0.380**	−**0.486**
Root (RT) dm		1	**0.704**	−0.121	−**0.416**
RAS dm			1	**0.537**	−0.214
RAS/RT ratio				1	0.145
Moisture					1

*Pearson coefficient's values in bold indicate significant correlation between parameters (p < 0.05)*.

### Pearl millet lines differentially impact diversity of bacterial communities in their rhizosphere

**Figure 3:**
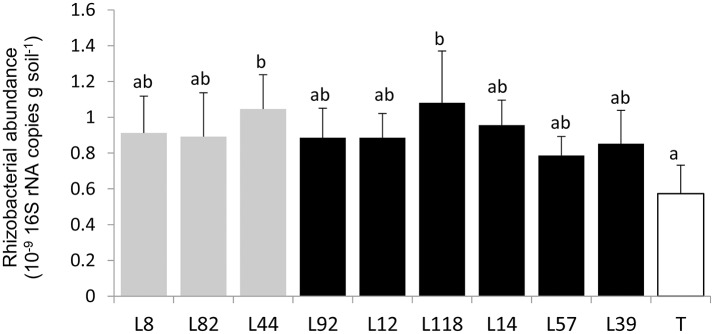
Quantification of rhizobacterial communities based on the number of 16S rRNA copies/g of rhizosphere soil of the 9 selected pearl millet lines and control soil (T) by q-PCR. Each bar plot represents mean of four replicates. The bars on the histogram represent the standard deviations Different letters indicate significant differences between millet lines according to Fischer LSD test (*p* < 0.05).

First, to quantify the bacterial communities in the root-adhering soil fraction (i.e., rhizosphere) of the 9 inbred lines, we measured the number of 16S rRNA gene copies per gram of soil using qPCR. There was no significant difference in bacterial abundance between pearl millet lines (*p* > 0.5) (Figure 3). There was a significant difference (*p* < 0.05) between bacterial abundance in the control unplanted soil (< 0.6 10^9^ 16S rRNA gene copies/g soil) and bacterial abundance in root-adhering soil of some millet lines (L44 and L118; respectively 1.04 × 10^9^ and 1.1 × 10^9^ 16S rRNA gene copies/g soil). Furthermore, mean bacterial abundance in rhizosphere soils was significantly increased compared to that of the control unplanted soils (0.8 × 10^9^ > 0.6 × 10^9^ copies/g soil).

DNA extracts from the root-adhering soil fraction of the 9 pearl millet inbred lines (four plants per line) were analyzed by PGM-based 16S rRNA gene diversity sequencing. We obtained 2.56 × 10^6^ sequences corresponding to 22,780 distinct OTUs, with an average of 64,072 sequences per sample. We used alpha diversity indexes to compare the total diversity of OTUs of the root-adhering soil fraction of the different pearl millet lines with the control unplanted soil. The number of analyzed sequences was 58,314 for control soil and ranged from 58,427 for root-adhering soil of line L92 to 72,849 for line L82. Alpha diversity metrics are summarized in Table 2 and comprise measures of richness based on qualitative (Chao1), OTUs count and quantitative (Shannon) estimators, as well as phylogeny-based measures such as phylogenetic diversity (PD_whole). The quantitative Shannon index indicates that α-diversity of the bacterial communities in the root-adhering soil fraction of the three pearl millet lines with low RAS/RT ratio (L8, L82, and L44) is significantly lower compared to the control unplanted soil (respectively *p* = 0.04, *p* = 0.02, and *p* = 0.02 with Kruskal-Wallis test). This is not the case for the six inbred lines with high RAS/RT ratio. For the three others calculated α–diversity indexes (PD_whole, Chao1, and OTUs count), we didn't find any significant difference between the different treatments and the control soil while they showed the same tendencies as that of the Shannon index (Table 2). The rarefaction curves for OTUs confirms that the number of OTUs estimated in the root-adhering soil fraction of the three pearl millet lines with low RAS/RT ratio is lower than the one of the six inbred lines with high RAS/RT ratio (Figure S2A). Globally, the number of estimated OTUs at 40,870 sequences depth in the root-adhering soil fraction of pearl millet lines (11,190 in average) is significantly lower (*p* = 0.03) than the one of the control unplanted soil (12,266 in average, Figure S2B).

**Table 2:** Bacterial Alpha diversity metrics calculated after data rarefaction analysis based on 40,870 sequences from each sample (9 pearl millet lines and the T control).

**Samples**	**PD_whole**	**Chao1**	**OTU**	**Shannon index**
L8	623 ± 14	32300 ± 9673	10341 ± 2385	^*^10.32 ± 0.48
L82	664 ± 23	34574 ± 1464	10836 ± 493	^*^09.89 ± 0.42
L44	643 ± 18	33676 ± 642	10548 ± 318	^*^10.04 ± 0.32
L92	668 ± 22	33935 ± 3047	11141 ± 339	10.59 ± 0.08
L12	734 ± 21	39013 ± 2693	12183 ± 368	10.83 ± 0.08
L118	650 ± 21	32865 ± 1533	10730 ± 404	10.38 ± 0.17
L14	699 ± 34	36703 ± 3983	11566 ± 617	10.71 ± 0.11
L57	686 ± 31	35028 ± 3529	11399 ± 555	10.58 ± 0.17
L39	723 ± 40	38834 ± 1205	11965 ± 111	10.71 ± 0.07
T	733 ± 39	38294 ± 4644	12266 ± 630	10.87 ± 0.16

* *indicates values that are significantly different from the control treatment value using Kruskal-Wallis test (p < 0.05)*.

**Figure 4:**
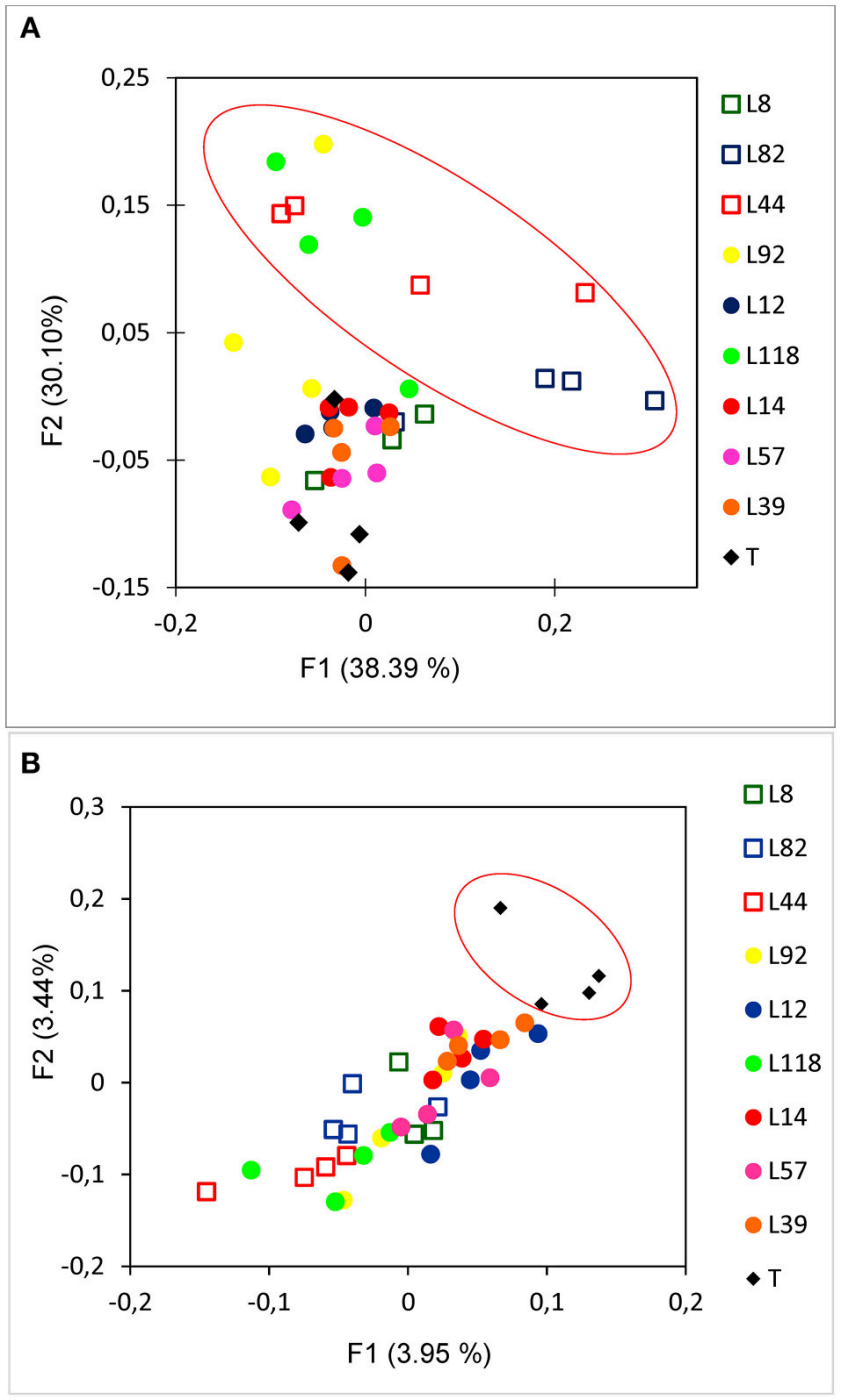
Principal Coordinate Analysis (PCoA) of weighted UniFrac **(A)** and Unweighted UniFrac **(B)** dispersion bacterial diversity of the 9 pearl millet inbred lines performed with 16S rRNA pyrosequencing. Low RAS/RT ratio lines are represented with square, high RAS/RT ratio lines are represented with circle.

In this work, we considered β-diversity to evaluate the differences in rhizosphere bacterial diversity between the 9 pearl millet lines. For that, we used weighted UniFrac distance metrics and a Principal Coordinate Analysis (PCoA) was performed to evaluate how the genetic traits of pearl millet lines could affect the structure of rhizobacterial communities and their relation with aggregation intensity estimated by the RAS/RT ratio (Figure 4A). There appeared to be a quite consistent clustering for the samples of each line. Most lines are close to each other in this projection, with the exception of 3 lines (L82, L44, and L118) clustering separately from the six other lines (*p* = 0.001; t = 7.16 using PERMANOVA, 999 permutations). But this separation is not obviously linked to the root-adhering soil phenotype (RAS/RT ratio), as the three inbred lines comprise two “low RAS/RT ratio” lines (L44 and L82) and 1 “high RAS/RT ratio” line (L118) (Figure 4A). Nevertheless, the PCoA and PERMANOVA performed on the unweighted UniFrac distance matrix that is sensitive to rare species (Lozupone and Knight, 2005) showed significant separation between rhizosphere soils and control soils (*p* = 0.001, t = 1.16 using PERMANOVA, 999 permutations) (Figure 4B).

### Pearl millet inbred lines significantly modulate the relative abundance of bacterial taxa (phyla and orders) in their rhizosphere

**Figure 5:**
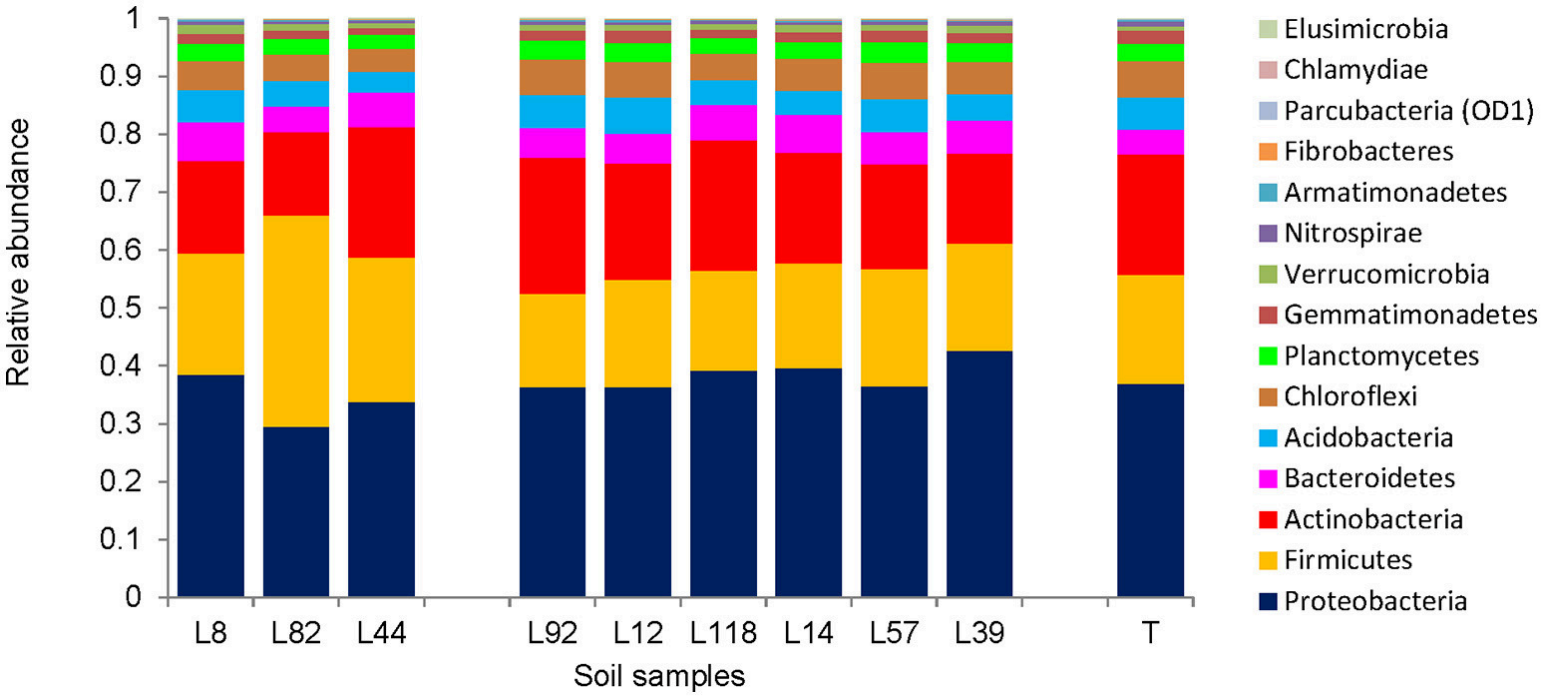
Distribution of 15 major bacterial phyla in the rhizosphere (root-adhering soil fraction) of the 9 pearl millet inbred lines and the control unplanted soil (T).

The bacterial composition of root-adhering soil fraction from the 9 contrasted pearl millet lines was examined at different taxonomic levels. At the phylum level, the bacterial microbiota composition in the root-adhering soil fraction of the 9 pearl millet lines was compared to that in the control unplanted soil (Figure 5). Among the 15 phyla detected in all fractions, the dominant ones (abundance > 20%) belonged to the *Proteobacteria, Firmicutes*, and *Actinobacteria*, and to a lesser extent to *Bacteroidetes, Acidobacteria, Chloroflexi, Planctomycetes*, and *Gemmatimonadetes* (1% < abundance < 20%) (Table S2). This molecular approach of the bacterial diversity revealed also three phyla found in all fractions but belonging to the “rare microbiosphere” (abundance < 0.1%): *Parcubacteria* (previously named OD1), *Chlamydiae*, and *Elusimicrobia* (Table S2). As a major overall tendency in observed bacterial communities at the phylum level, the three pearl millet lines with low RAS/RT ratio (L8, L82, and L44) taken together exhibited a lower abundance of *Proteobacteria* (33.9 vs. 38.4%, *p* < 0.02) and a higher abundance of *Firmicutes* (27.5 vs. 18.1%, *p* < 0.002) when compared (using Kruskal-Wallis test) to the lines with high RAS/RT ratio. However, within the two groups there was some heterogeneity in distribution, so that taken individually, not every line in the low RAS/RT group was different from every line in the high RAS/RT group. For instance, in L8 rhizosphere, the relative abundances of *Proteobacteria* (38.4%) and *Firmicutes* (21.1%) were not significantly different from those of the high ratio lines (*p* > 0.05; Kruskal-Wallis test). Moreover, three lines among the six with high RAS/RT ratio (L92, L12, and L57) did not show significant difference for *Proteobacteria* compared to the low ratio lines. A statistical analysis (Kruskal-Wallis) was applied to compare phylum proportions between each line and the control unplanted soil (Table S2).

**Figure 6:**
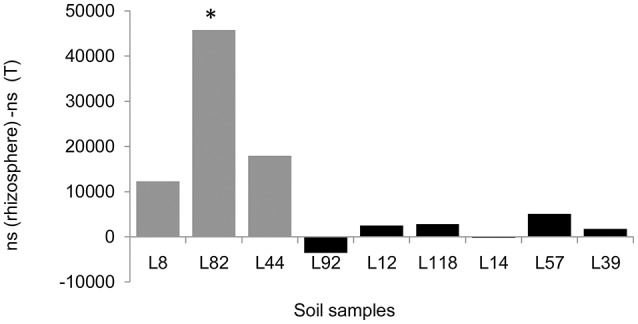
Difference in mean abundance of Bacillales (*ns* = number of sequences) between rhizosphere soil of each pearl millet inbred lines and the control unplanted soil (T). ^*^Indicates significant difference in sequences counts using Kruskal-Wallis test (*P* < 0.05).

At the order level, the most abundant ones are *Bacillales* (*Firmicutes*), *Rhizobiales* (*Proteobacteria*), and *Actinomycetales* (*Actinobacteria*), these three orders representing 60–70% of bacterial diversity, in agreement with data observed at the phylum level (Table S3). When compared to lines with low RAS/RT ratio, the mean percentage of *Bacillales* in rhizospheric soil of lines with high RAS/RT ratio was significantly lower (27.6 vs. 39.8%, *t*-test *p* < 0.05) and the mean percentage of *Rhizobiales* significantly higher (22.2 vs. 17.4%, *t*-test *p* < 0.02) (Table S3). Like what was observed at phylum level, these overall tendencies could change if we compare individually different lines to each other using Kruskal-wallis and Dunn pairwise comparison tests. Indeed, only L82 line showed significant difference on *Bacillales* relative abundance compared to L118 and L92 millet lines. For *Rhizobiales*, significant differences were found between Low RAS/RT lines L82 and L44 and the high RAS/RT lines L39 and L14 (Table S3). Concerning *Bacillales*, these contrasts on relative abundance also translate into significant differences in numbers of sequences between root-adhering soil fractions from L82 and that from the control soil as illustrated in Figure 6. For *Rhizobiales*, no significant difference was found in sequences count between treatments.

**Figure 7:**
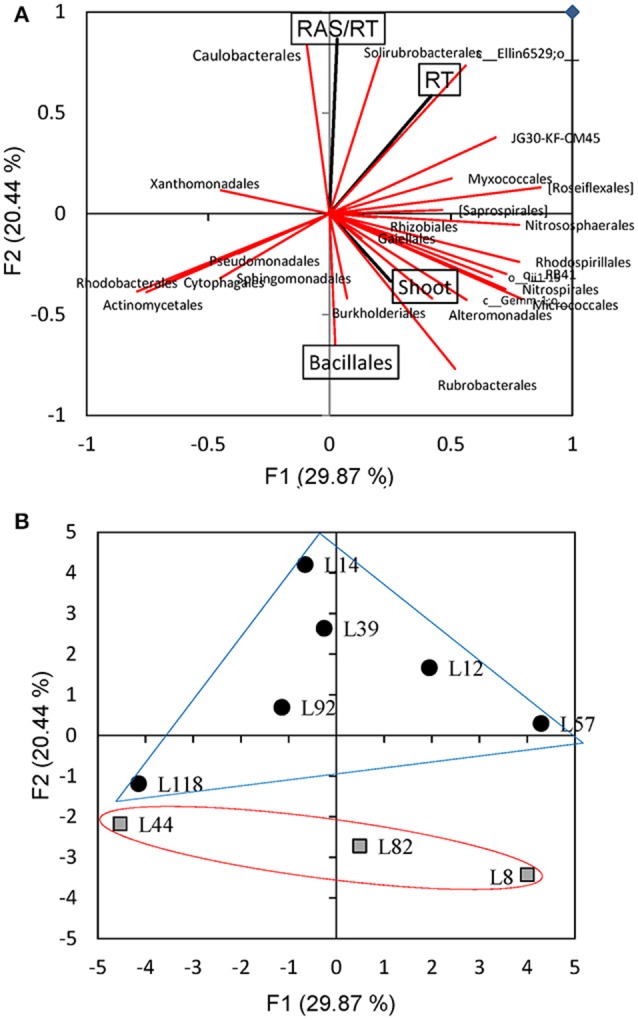
Principal component analysis of RAS/RT, shoot dm, root dm, and abundances of the 26 main bacterial orders found in the rhizosphere of 9 pearl millet inbred lines. **(A)** Plot of the loadings of the variables with principal components F1 and F2. **(B)** Projection in F1^*^F2 plan of rhizosphere soil samples from the different millet lines.

A Principal Component Analysis was performed to analyse the relations between the frequency of the 26 most abundant bacterial orders and plant parameters (RAS/RT ratio, shoot, and root dry mass). The variance explained by the two principal components PC1 and PC2 is 50.3% (Figure 7A). The PC2 explaining 20.4% of the variance had a positive contribution of parameter RAS/RT (12.6%), *Caulobacterales* frequency (11.7%), and *Solirubrobacterales* frequency (10.2%) of and a negative contribution of *Bacillales* (−7.1%) and *Rubrobacterales* (−10.0%) frequencies. This data analysis confirmed the negative correlation between abindance of one of the dominant bacterial orders (*Bacillales*) and RAS/RT ratio (Figure 6). The three other orders showing positive (*Caulobacterales, Solirubrobacterales*) or negative (*Rubrobacterales*) correlation with RAS/RT ratio, are not abundant in our samples (3–4, 1–3%, < 1% of sequences, respectively). The negative correlation previously described between RAS/RT and shoot dry mass also appears in the PCA plot. When we further plot the observations on this PCA coordinates space, there is a separation along the second axis between the 3 lines with low RAS/RT ratio (L8, L82, L44) and lines with high RAS/RT ratio (L92, L12, L14, L57, L39) (Figure 7B). Along this PC2 axis, the L118 line (high RAS/RT ratio) appears again to be in an intermediate position (Figure 7B).

### Pearl millet inbred lines significantly modulate the relative abundance of cultivable bacterial species in their rhizosphere, including EPS-producing species

**Figure 8:**
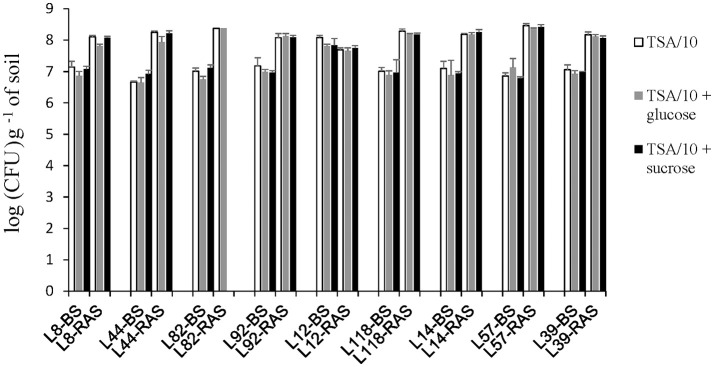
Enumeration of bacterial microbiota on TSA/10 (in white). TSA/10 + glucose (in gray) and TSA/10 + sucrose (in black) expressed in colony-forming units (CFU) per gram of bulk soil (-BS) or root-adhering soil (-RAS). Missing data for the treatment L82.

Whatever the medium used for cultivable bacteria numeration, the average count in the soil not adhering to the roots was about 1 × 10^7^ CFU per gram (Figure 8). In the root-adhering soil fraction, a 10-fold average increase of cultivable bacteria abundance was observed in 8 out the 9 inbred lines (mean value 1.2 × 10^8^, *p* < 0.001). The only exception was line L12 for which the counting in bulk soil is not significantly different from counting in root-adhering soil (Figure 8). The two counts were higher than those recorded for bulk soil in the other inbred lines, suggesting a rhizosphere effect extending beyond the root-adhering zone in L12 (Figure 8). No significant difference was detected between abundances of cultivable bacteria in the rhizosphere of these 9 pearl millet lines. Neither did addition of glucose or sucrose significantly influence numeration results.

Based on their mucoid phenotype, we isolated 184 EPS-producing bacterial strains from TSA/10 medium supplemented with glucose or sucrose. These strains were identified at the species level using PCR-sequencing of 16S rRNA gene (Table S4). Among the 48 identified species (Table 3), the most frequent ones belonged to *Actinobacteria* (*Arthrobacter pascens*: 28 strains, *Arthrobacter globiformis*: 13 strains, *Microbacterium barkeri*: 30 strains), *Firmicutes* (*Bacillus aryabhattai*: 22 strains, *Paenibacillus polymyxa*: 16 strains), and *Proteobacteria* (*Pseudomonas plecoglossicida*: 13 strains). These data are in agreement with the data presented at the phylum and order levels. To evaluate the quantitative contribution of the most frequent EPS-producing species, we selected 18 species (Table 4), belonging to *Actinobacteria, Firmicutes*, and *Proteobacteria*, with 16S rRNA sequences > 700 bp to optimize the taxonomic assignment and we performed Blast analysis against our OTU database (Table 4). We didn't observe striking differences between the two groups of pearl millet lines (low and high RAS/RT ratio). The low RAS/RT ratio L44 line is characterized by a high increase of bacterial sequences corresponding to *Actinobacteria* (three species of *Arthrobacter* and one of *Microbacterium*) and *Proteobacteria* (*Ochrobactrum pseudogrignonense, Rhizobium pusense*, and *Variovorax paradoxus*) compared to control unplanted soil. We also found for low RAS/RT ratio L82 line an increase for two species of *Bacillus* (Firmicutes), and in low RAS/RT ratio L8 line an increase for two species of *Proteobacteria* (*R. pusense* and *V. paradoxus*) (Table 4). For high RAS/RT ratio lines, three *Arthrobacter* species were significantly increased in two out of the 6 lines (L92, L118), and none species belonging to *Firmicutes*. Significant increase of sequences fitting with the one of *R. pusense* was detected in the rhizosphere of high RAS/RT ratio L118 and L14 lines. Line 118 greatly differed from the other lines with high RAS/RT as it exhibited significant increase in number of sequences of *Arthrobacter* (three species), *Pseudomonas* (two species), and *R. pusense* (Table 4). Sequences of *V. paradoxus* (Table 4) were indifferently detected in the rhizosphere of lines with low- (L8, L44) and high RAS/RT ratio (L14, L39, L57).

**Table 3:** Distribution of dominant bacterial EPS-producing species isolated on C-enriched TSA/10 media from the rhizosphere (root-adhering soil fraction) of 9 pearl millet inbred lines.

**Strains**	**L8**	**L82**	**L44**	**L92**	**L12**	**L118**	**L14**	**L57**	**L39**	**Total**
*A. chlorophenolicus*	0	0	0	0	2	0	0	0	1	3
*Arthrobacter defluvii*	0	0	0	0	6	0	0	1	0	7
*Arthrobacter globiformis*	0	1	2	1	0	1	4	1	3	13
*Arthrobacter pascens*	0	2	3	1	13	1	3	0	5	28
*Microbacterium barkeri*	2	0	9	1	3	4	3	2	6	30
*Bacillus aryabhattai*	0	3	1	2	9	0	2	3	2	22
*Paenibacillus polymyxa*	1	0	2	4	1	0	6	2	0	16
*Enterobacter xiangfangensis*	0	1	0	0	0	0	2	0	0	3
*O. pseudogrignonense*	0	0	0	0	0	0	0	0	5	5
*Pseudomonas monteilii*	1	0	0	1	0	0	0	0	1	3
*P. plecoglossicida*	0	0	3	4	0	0	5	0	1	13
*Rhizobium pusense*	0	0	0	0	0	0	1	1	1	3
Total	4	7	20	14	34	6	26	10	25	146

*A, Arthrobacter; P, Pseudomonas; O, Ochrobacterium*.

**Table 4:** Blast of isolates sequences on PGM sequencing OTU table database.

**Strains**	**OTUs**	**Bit score**	**L8**	**L82**	**L44**	**L92**	**L12**	**L118**	**L14**	**L57**	**L39**	**T**
*Arthrobacter pascens/globiformis*	49	[357;490]	603.75 a	632.5 a	**2,146.25 b**	**1,604 b**	711.5 a	**1,836.5 b**	294 a	375.5 a	252.75 a	83.75 a
*Arthrobacter crystallopoietes*	48	[399;483]	460.25 ab	601.25 ab	**1,238.75 c**	**930 bc**	614 ab	**1,345 c**	311.75 a	363.5 ab	256.75 a	148 a
*Arthrobacter ureafaciens*	2	[457;479]	33 ab	**77.5 bc**	**152 de**	**110 cd**	39.5 ab	**217.5 e**	29.5 ab	42.25 abc	24.5 ab	3.25 a
*Bacillus megaterium NBRC 15308*	59	[427;484]	1,168.5 a	2671.75 a	1,295.25 a	562.75 a	737.25 a	825.5 a	595.5 a	698 a	583.75 a	431 a
*Bacillus megaterium/arabhattai*	86	[431;494]	1,959.25 a	**4,602.5 b**	2,216.5 a	938 a	1,240.25 a	1321 a	1,014.75 a	1,170.25 a	991.25 a	741.25 a
*Bacillus tequilensis*	8	[448;488]	42.25 ab	**91.25 b**	52.75 ab	68.5 ab	51.5 ab	78.5 ab	61 ab	46.5 ab	28.25 a	33.25 a
*Paenibacillus polymyxa SC2*	4	[466;486]	49.25 ab	18.25 ab	55.25 b	5.75 a	17.25 ab	16 ab	10.75 ab	8.75 a	16.75 ab	13.5 ab
*Paenibacillus polymyxa E681*	4	[357;381]	41 ab	16.25 ab	50.75 b	4.75 a	15.75 ab	14 ab	9.5 ab	6.5 a	15 ab	11.5 ab
*M. paraoxydans/barkeri*	1	407	1 ab	1.25 ab	**2.5 b**	1.25 ab	1.75 ab	1.25 ab	2.25 ab	1.5 ab	0.75 ab	0.5 a
*Exigobacterium indicum*	1	460	0.75 a	0.75 a	1 a	0.5 a	4.5 a	1 a	0 a	0 a	0.25 a	0.25 a
*Enterobacter xiangfangensis*	2	[460;494]	7 a	36.25 ab	15.5 a	105.25 b	8.75 a	26.5 a	4.25 a	1.75 a	29 a	3.5 a
*Pseudomonas monteilii*	9	[422;488]	94 ab	34.75 ab	19 ab	54.25 ab	44.5 ab	**321.25 b**	7.25 a	4 a	126 ab	4 a
*Ochrobactrum pseudogrignonense*	2	[466;499]	3 a	0 a	**20.5 b**	0.25 a	1.5 a	1.75 a	1.75 a	0 a	1.25 a	0.25 a
*Stenotrophomonas pavanii*	2	[473;483]	5.25 ab	6.5 ab	**14.25 b**	9 ab	9.5 ab	11.25 ab	0 a	2.25 a	5.25 ab	1 a
*Pseudomonas plecoglossicida*	10	[427;494]	104.75 ab	35.5 ab	42.75 ab	57.25 ab	48 ab	**328.25 b**	14.5 a	4.5 a	133.75 ab	4.5 a
*Variovorax paradoxus S110*	5	[327;470]	**29 cd**	9 ab	**31 d**	9.25 ab	9.75 ab	16 abc	**19.25 bcd**	**19.25 bcd**	**23.5 cd**	4 a
*Rhizobium pusense/A. fabrum*	6	[466;488]	**105.5 bc**	81.5 ab	**163.25 cd**	84.25 ab	98.25 abc	**220.5 d**	**102 bc**	64.25 ab	81.5 ab	27.25 a
*Corynebacterium ilicis*	8	[366;490]	44.75 ab	102 bc	**201.5 de**	155.5 cd	53.75 ab	**296.5 e**	39 ab	58 ab	32.5 ab	3.75 a

*Reported numbers of OTUs are those that matched the isolates sequences at 97% threshold. Column 2 (“OTUs”) indicates the count of matched OTUs in the database. Means of estimated abundance are recorded in column 4–13 for rhizosphere soils of pearl millet inbreed lines and the control soil (T). Different letters indicate significant differences between samples in the same row (Fisher LSD test p < 0.05). The bold frames indicate the values significantly higher than in control soil. M, Microbacterium; A, Agrobacterium*.

## Discussion

We have shown that RAS/RT ratio in pearl millet is strongly dependent on plant line and therefore under genetic control. But besides plant species (de León-González et al., 2006) and intraspecific variability (our study), the RAS/RT ratio depends on several other factors such as harvesting conditions, plant development stage, and soil moisture (Alami et al., 2000). We designed plant growth conditions to minimize variations in soil moisture. Accordingly, no significant correlation was found in this study between RAS/RT ratio and soil moisture, so that this parameter did not constitute a major factor of variation in our case. The separation between root-adhering soil and bulk soil was carried out as uniformly as possible using mechanical shaking. In these conditions, the genetic traits of plant were the main putative factor that could explain the differences recorded on this parameter between lines. Therefore, this result confirms recent results in literature that reported a genotypic variation in the size of rhizosheath in wheat (Delhaize et al., 2012) and barley (George et al., 2014). The first objective of our study was reached with the ranking of pearl millet lines based on the RAS/RT ratio, and two groups were constituted in a final subset of 9 selected lines: a group with low RAS/RT ratio (L8, L82, and L44) and a group with high RAS/RT ratio (L92, L12, L118, L14, L57, and L39) (Figures 1, 2). The separation between inbred lines of the main groups (high and low ratios) was always conserved. However, average values of the RAS/RT ratio, amplitudes in differences and the ranking between lines within groups could vary depending of the phenotyping experiments. We hypothesized two sources of variability: plant growth conditions and the methodology used for collecting the root-adhering soil fraction. The latter method was previously used for various soils, cambric arenosol (Gouzou et al., 1993), eutric cambisol (Bezzate et al., 2000), dystric cambisol (Alami et al., 2000), clay-silty soil (Kaci et al., 2005), and vertisol (Amellal et al., 1998). These soils contained from 12 to 55% clay. In our case, soils containing less than 3% clay, root-adhering soil amounts after shaking are limited, hence it possibly induced higher relative variance for this measurement. Concerning plant growth conditions, we have seen that substantial differences could occur between blocks in the screening experiment. As these blocks were conducted sequentially, variations in environmental conditions like light and temperature might explain a large fraction of the observed variability. The ranking obtained in the 3rd experiment, where the 9 lines were sown together in a single block and with a larger number of repeats, is certainly the most robust. But again, the separation between the most contrasted phenotypes was always found whatever the experimental conditions. Concerning the phenotypic variation range, RAS/RT ratio varied between 6 and 35 (g/g) in our study whereas in barley phenotyping, George et al. (2014) found higher values, with variation ranging from 20 up to 200 (g/g). This could be explained by several sources of variation, relying on experimental protocol (differences in clay content between soils, phenological stage at harvest, etc.) and also on differences in root structures (root hair length for instance) and physiology between the two cereals (Brown et al., 2012).

Several biological determinants of soil aggregation can be envisioned such as root hair characteristics (Moreno-Espíndola et al., 2007; Brown et al., 2012), mycorrhizae (Wu et al., 2014, 2015; Rillig et al., 2015), and rhizodeposition-mediated interaction with EPS-producing soil bacteria. In our study we focused on root influence on bacterial communities. As an assay of bacterial abundance, the number of 16S rRNA gene copies per gram of soil was significantly higher in the rhizosphere (root-adhering soil fraction) of pearl millet lines compared to control unplanted soil, as found in previous studies (Kielak et al., 2008) (Figure 3). However, there was no variation between lines of this number of 16S rRNA gene copies/g of rhizospheric soil, there again in agreement with previous data (Aira et al., 2010) on maize. In line with these observations, the abundance of cultivable bacteria was 10 times higher in root-adhering soil fractions compared to control unplanted soil, and quite similar between lines. Accordingly, it was observed that different maize accessions did not exhibit significant differences in numbers of CFU counts in their rhizosphere soil (Chiarini et al., 1998).

Alpha diversities are impacted by plant root vicinity, being lower in rhizospheric soil than in bulk soil. Further, α-diversity around roots decreased in millet lines with low RAS/RT ratio. This result supports the effect of plant root exudation on the rhizobacterial community selection (Paterson et al., 2007; Peiffer et al., 2013; Mendes et al., 2014). The fact that this reduction of bacterial alpha diversity in the rhizospheres differed between lines may be linked to differences in their root exudation patterns (Czarnota et al., 2003; Micallef et al., 2009).

Bacterial β-diversity is also affected by the presence of plant roots (Lundberg et al., 2012; Peiffer et al., 2013; Panke-Buisse et al., 2015). However, in our study, using weighted UniFrac metrics based on the entire OTUs record, rhizospheric soil samples from the majority of our lines (6 out of 9) were not separated from control soil samples. This is due to the fact that the diversity of the most abundant species is not strongly affected by millet root variability in our conditions. Further, for the three lines which seemed to cluster at some distance from control soil in this analysis, there was no correlation with the soil aggregation patterns. Conversely, we observed clear separations between control soils and rhizospheric soils using unweighted UniFrac distance analysis, suggesting differences in diversity of rare species as previously reported in Lozupone and Knight (2005). But there again, clustering was not obviously linked with soil aggregation patterns, although some grouping between samples from lines with low RAS/RT could be visualized (Figure 4B).

Metabarcoding allowed demonstration of an increase in *Firmicutes* (*Bacillales*) and a decrease in *Proteobacteria* (*Rhizobiales*) frequencies with 2 among the 3 lower RAS/RT ratio lines. On the other hand, the most frequent and dominant EPS-producing cultivable bacterial species we have isolated belonged to *Arthrobacter* and *Microbacterium* genera (*Actinobacteria*), *Bacillus* and *Paenibacillus* genera (*Firmicutes*) and *Pseudomonas, Ochrobactrum*, and *Rhizobium* genera (*Proteobacteria*). *Paenibacillus polymyxa* strains (Gouzou et al., 1993; Bezzate et al., 2000; Guemouri-Athmani et al., 2000) or EPS-producing *Rhizobium* species (Alami et al., 2000; Kaci et al., 2005) have been shown to be associated with root soil aggregation in previous studies. Interestingly in our study, we have identified two strains belonging to *Proteobacteria* phylum that were exclusively isolated from rhizosphere of high aggregation lines: *O. pseudogrignonense* in L39 rhizosphere soil and *R. pusense* in L14, L57, and L39 rhizosphere soils.

However, these tendencies were not recovered by the coupling of culture dependent and independent method. Indeed, if we simply look for abundance in NGS records of OTUs showing homology with 16S sequence of isolated EPS-producing strains, we did not identify species which, putatively, could be specifically linked with high RAS/RT ratio or low RAS/RT ratio. We found significant enrichment in OTUs homologs of 12 (out of 18) EPS-producing strains in rhizospheric vs. control soil samples for several pearl millet lines (Table 4), but this enrichment was not obviously linked with variations in RAS/RT ratio. Of course we could not rule out that some other (non-isolated) species might contribute to soil aggregation diversity. Moreover, one cannot exclude that the mechanisms concurring to soil aggregation in a given line are not exactly the same in another line. This may include factors linked to other biological factors than bacterial activities. A great space is still open for further studies to decipher the precise underlying mechanisms.

## Conclusion

We found a significant variability in rhizospheric soil aggregation associated with plant genetic diversity. The diversity between inbred lines is also associated with variations in bacterial community composition. It would now be worth assessing how variable the selected millet lines may be in terms of root exudates amount and composition, and particularly to check whether phenotypic variations are correlated to production of specific substrates. The occurrence of such plant genetic variability offers opportunity to assess if it might be an interesting target for crop selection. In a context of agroecological cropping systems, harnessing positive interactions between plants and soil communities might be interesting to sustain production in a challenging environment.

## Author contributions

PN, NK, YV, KA, IN, TH, and LC designed the study. PN, MG, AP, DD, and MBH performed the experiments. PN, PO, and MB performed bioinformatics analysis. PN, YV, LCL, WA, TH, and LC wrote the paper.

### Conflict of interest statement

The authors declare that the research was conducted in the absence of any commercial or financial relationships that could be construed as a potential conflict of interest.
