# Supplementary material for: Pearl Millet Genetic Traits Shape Rhizobacterial Diversity and Modulate Rhizosphere Aggregation
Source: Front Plant Sci. 2017 Jul 27;8:1288. doi: 10.3389/fpls.2017.01288 (PMC5529415; doi:10.3389/fpls.2017.01288)
Supplement: Supplementary file 1 [file DataSheet1.PDF]

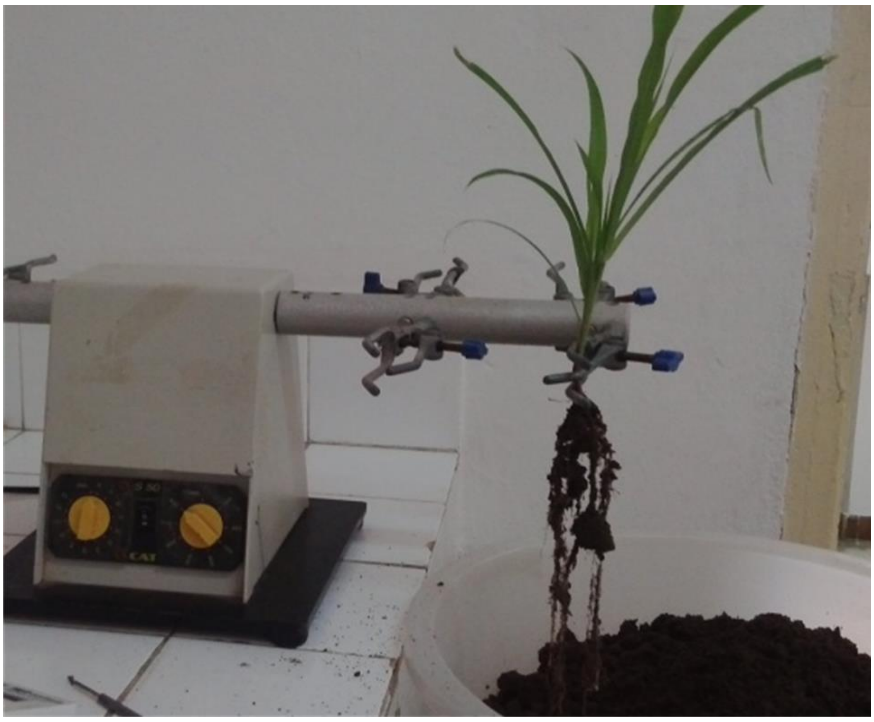

Figure S1: Illustration of setup used for plant shaking and RAS recovery.

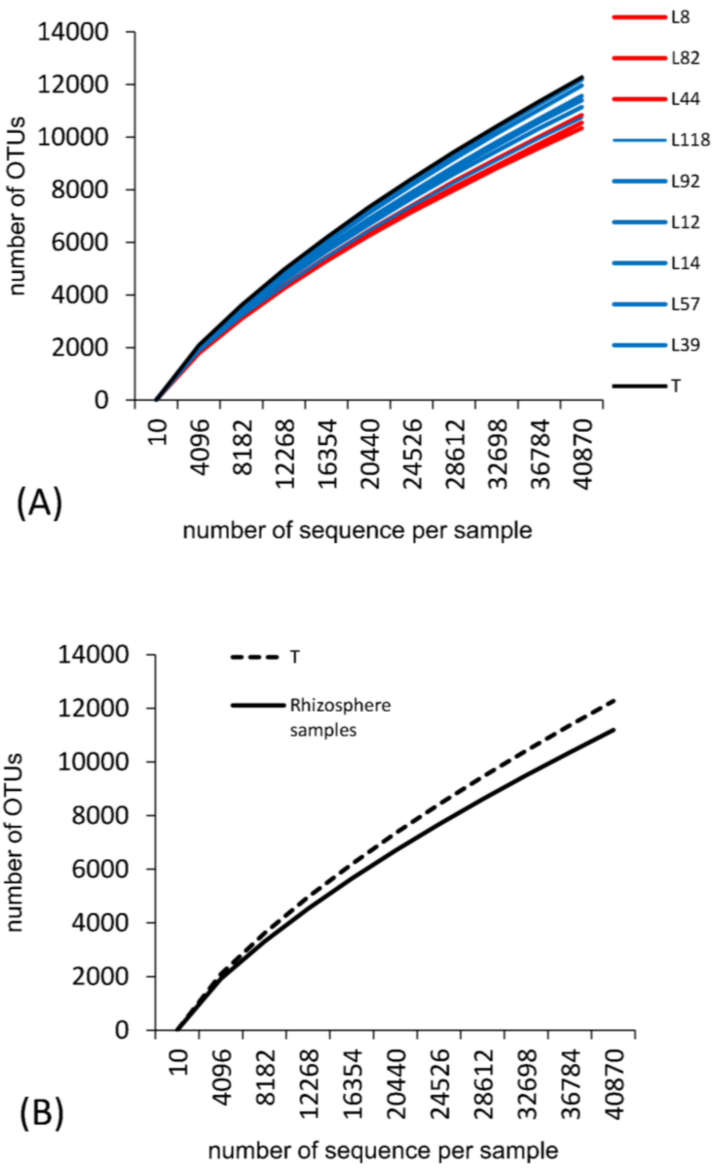

Figure S2: (A) Bacterial OTU rarefaction curves of the root-adhering soil of 9 pearl millet inbred lines (per group of the RAS/RT ratio values used to select the 9 millet lines). **(B)** bacterial OTU rarefaction curves of millet rhizosphere samples *vs* control unplanted soil (T).

Table S1: Values of RAS dm, RAS/RT ratio, bulk soil moisture, shoot and root dm raw data after harvesting of pearl millet plants during the phenotyping experiment (86 pearl millet inbred lines). Data are gathered by block.

Block	Line	RAS dm (g)	RAS/RT	Bulk Soil moisture (%)	Shoot dm (g)	Root dm (g)
S1	L1	0.698	18.17	6.29	0.212	0.038
S1	L1	0.358	11.18	6.62	0.203	0.032
S1	L1	0.101	10.13	9.27	0.058	0.010
S1	L1	0.224	20.72	9.99	0.065	0.011
S1	L1	0.501	15.65	6.19	0.124	0.032
S1	L5	0.202	8.07	6.93	0.099	0.025
S1	L5	0.466	13.25	7.52	0.213	0.035
S1	L5	0.368	7.28	4.10	0.243	0.051
S1	L5	0.105	5.85	5.57	0.097	0.018
S1	L5	0.439	7.27	7.58	0.180	0.060
S1	L6	0.659	20.41	8.59	0.135	0.032
S1	L6	0.750	13.32	8.35	0.113	0.056
S1	L6	0.276	16.34	6.59	0.076	0.017
S1	L6	0.783	15.38	6.98	0.221	0.051
S1	L6	0.826	19.44	5.28	0.169	0.043
S1	L7	0.122	9.42	6.45	0.127	0.013
S1	L7	0.905	14.30	4.63	0.342	0.063
S1	L7	0.818	14.51	4.04	0.368	0.056
S1	L7	0.332	10.57	6.20	0.202	0.031
S1	L7	0.487	8.28	5.46	0.255	0.059
S1	L8	0.215	7.58	6.77	0.218	0.028
S1	L8	0.161	8.20	8.62	0.067	0.020
S1	L8	0.113	2.82	4.79	0.197	0.040
S1	L8	0.230	7.11	5.54	0.191	0.032
S1	L8	0.135	8.98	6.68	0.154	0.015
S1	L9	0.488	14.82	6.23	0.170	0.033
S1	L9	0.365	10.42	5.01	0.174	0.035
S1	L9	0.106	10.84	8.84	0.047	0.010
S1	L9	0.232	10.40	7.47	0.078	0.022
S1	L9	0.095	10.29	5.63	0.052	0.009
S1	L14	0.666	24.12	7.58	0.051	0.028
S1	L14	0.570	19.99	8.30	0.138	0.029
S1	L14	0.551	17.88	9.28	0.120	0.031
S1	L14	0.639	22.42	6.67	0.110	0.029
S1	L14	0.653	16.40	6.94	0.137	0.040
S1	L20	1.712	21.27	4.43	0.406	0.081
S1	L20	0.998	19.37	4.75	0.367	0.052
S1	L20	0.719	13.64	4.53	0.391	0.053
S1	L20	1.341	10.45	3.15	0.370	0.128

S1	L20	1.047	16.56	3.56	0.365	0.063
S1	L10	0.384	12.01	5.88	0.246	0.032
S1	L10	1.045	11.40	3.74	0.309	0.092
S1	L10	1.181	11.07	3.35	0.295	0.107
S1	L10	1.241	9.46	6.58	0.208	0.131
S1	L10	1.191	13.31	5.99	0.242	0.090
S1	L12	0.594	18.85	8.06	0.137	0.032
S1	L12	1.665	19.29	5.06	0.236	0.086
S1	L12	0.828	20.66	5.99	0.158	0.040
S1	L12	0.331	23.96	7.99	0.068	0.014
S1	L12	0.894	35.19	6.25	0.232	0.025
S1	L11	0.312	16.43	8.53	0.200	0.019
S1	L11	0.193	10.32	3.85	0.158	0.019
S1	L11	0.559	13.01	5.46	0.190	0.043
S1	L11	1.232	14.61	5.38	0.216	0.084
S1	L11	0.169	10.01	7.95	0.117	0.017
S1	L18	0.121	6.49	10.97	0.013	0.019
S1	L18	0.293	12.95	6.94	0.121	0.023
S1	L18	0.289	13.61	7.45	0.031	0.021
S1	L18	0.068	1.50	6.38	0.031	0.045
S1	L18	0.095	5.00	6.35	0.022	0.019
S2	L25	0.128	12.8	14.14	0.116	0.010
S2	L25	0.447	17.89	20.47	0.218	0.025
S2	L25	0.390	11.15	9.68	0.175	0.035
S2	L25	0.535	14.08	8.65	0.279	0.038
S2	L27	0.103	12.88	16.25	0.090	0.008
S2	L27	0.177	11.82	16.81	0.093	0.015
S2	L27	0.245	16.30	18.56	0.183	0.015
S2	L27	0.200	19.96	9.77	0.158	0.010
S2	L27	0.381	14.67	9.6	0.203	0.026
S2	L23	0.381	18.15	15.91	0.188	0.021
S2	L23	0.325	16.27	13.89	0.152	0.020
S2	L23	0.269	17.94	13.5	0.185	0.015
S2	L23	0.448	10.43	7.28	0.249	0.043
S2	L23	0.263	11.94	12.63	0.236	0.022
S2	L31	0.019	3.84	15.44	0.027	0.005
S2	L31	0.050	16.79	17.94	0.030	0.003
S2	L31	0.342	12.23	20.62	0.246	0.028
S2	L31	0.153	11.79	11.41	0.145	0.013
S2	L31	0.056	9.28	10.95	0.060	0.006
S2	L32	0.172	9.56	12.5	0.078	0.018
S2	L32	0.113	5.66	10.91	0.076	0.020
S2	L32	0.177	9.83	9.88	0.088	0.018
S2	L32	0.061	6.78	11.37	0.074	0.009
S2	L32	0.121	12.07	12.58	0.064	0.010
S2	L33	0.287	9.88	18.85	0.153	0.029

S2	L33	0.253	12.65	20.03	0.151	0.020
S2	L33	0.129	8.06	11.72	0.119	0.016
S2	L33	0.167	13.91	11.28	0.102	0.012
S2	L33	0.263	13.17	7.74	0.135	0.020
S2	L28	0.154	9.64	9.41	0.103	0.016
S2	L28	0.400	14.8	11.38	0.246	0.027
S2	L28	0.027	6.70	12.72	0.022	0.004
S2	L28	0.068	11.3	13.32	0.011	0.006
S2	L29	0.192	19.2	18.47	0.100	0.010
S2	L29	0.235	12.37	9.07	0.117	0.019
S2	L29	0.305	10.88	10.96	0.171	0.028
S2	L29	0.219	11.52	11.92	0.114	0.019
S2	L82	0.284	16.68	12.3	0.212	0.017
S2	L82	0.208	14.84	13.29	0.106	0.014
S2	L82	0.194	14.94	14.18	0.132	0.013
S2	L82	0.142	8.36	9.16	0.096	0.017
S2	L111	0.415	18.86	13.70	0.123	0.022
S2	L111	0.181	10.66	10.70	0.080	0.017
S2	L111	0.341	11.76	11.98	0.170	0.029
S2	L111	0.175	10.91	11.07	0.092	0.016
S2	L114	0.171	13.16	13.35	0.077	0.013
S2	L114	0.105	10.51	13.92	0.056	0.010
S2	L114	0.135	9.66	17.58	0.063	0.014
S2	L114	0.073	9.08	17.97	0.048	0.008
S2	L114	0.171	13.17	12.72	0.074	0.013
S2	L35	0.114	14.26	15.82	0.062	0.008
S2	L35	0.229	12.73	19.89	0.112	0.018
S2	L35	0.269	9.59	11.98	0.118	0.028
S2	L35	0.198	4.40	8.89	0.075	0.045
S2	L35	0.335	16.73	10.00	0.110	0.020
S3	L53	0.796	14.88	7.48	0.392	0.054
S3	L53	0.147	11.6	9.55	0.100	0.013
S3	L53	0.122	17.44	6.88	0.108	0.007
S3	L53	0.090	9.41	8.73	0.088	0.010
S3	L53	0.248	13.38	9.48	0.087	0.019
S3	L43	0.312	16.49	5.72	0.157	0.019
S3	L43	0.431	14.86	5.33	0.186	0.029
S3	L43	0.560	18.35	8.13	0.194	0.031
S3	L43	0.297	15.99	6.52	0.156	0.019
S3	L43	0.362	11.6	6.53	0.209	0.031
S3	L47	0.190	12.93	10.23	0.096	0.015
S3	L47	0.044	8.90	10.79	0.083	0.005
S3	L47	0.287	9.97	7.03	0.114	0.029
S3	L47	0.277	16.60	7.15	0.095	0.017
S3	L47	0.290	18.86	8.01	0.124	0.015
S3	L37	0.266	11.72	7.42	0.140	0.023

S3	L37	0.594	15.03	4.56	0.188	0.040
S3	L37	0.353	10.96	7.28	0.110	0.032
S3	L37	0.525	11.65	4.6	0.240	0.045
S3	L37	0.278	14.32	7.33	0.159	0.019
S3	L48	0.122	11.95	9.29	0.085	0.010
S3	L48	0.812	17.61	4.05	0.469	0.046
S3	L48	0.605	12.1	7.05	0.446	0.050
S3	L48	0.187	12.64	8.15	0.082	0.015
S3	L48	0.517	14.40	6.37	0.289	0.036
S3	L45	0.305	10.00	7.48	0.217	0.031
S3	L45	0.481	17.25	10.28	0.272	0.028
S3	L45	0.665	17.82	6.87	0.193	0.037
S3	L45	0.560	14.25	7.58	0.179	0.039
S3	L45	0.873	18.04	7.64	0.197	0.048
S3	L38	0.324	17.43	9.78	0.108	0.019
S3	L38	0.230	14.30	6.75	0.106	0.016
S3	L38	0.212	7.81	6.48	0.173	0.027
S3	L38	0.195	17.54	4.79	0.085	0.011
S3	L38	0.495	14.55	6.21	0.269	0.034
S3	L50	0.382	11.34	5.21	0.140	0.034
S3	L50	0.462	11.59	7.25	0.153	0.040
S3	L50	0.450	15.42	5.39	0.178	0.029
S3	L50	0.349	9.86	5.78	0.164	0.035
S3	L50	0.431	16.95	7.58	0.090	0.025
S3	L41	0.295	8.50	9.86	0.171	0.035
S3	L41	0.796	11.17	4.50	0.295	0.071
S3	L41	1.162	14.84	5.08	0.298	0.078
S3	L41	0.281	19.51	7.51	0.185	0.014
S3	L41	0.292	21.78	7.43	0.093	0.013
S3	L44	0.180	7.58	10.00	0.158	0.024
S3	L44	0.189	9.96	9.58	0.130	0.019
S3	L44	0.151	10.05	7.31	0.092	0.015
S3	L44	0.218	8.76	8.88	0.126	0.025
S3	L44	0.126	7.95	9.29	0.084	0.016
S3	L46	0.784	22.6	6.02	0.178	0.035
S3	L46	0.386	11.07	4.89	0.300	0.035
S3	L46	0.609	15.30	6.21	0.245	0.040
S3	L46	0.530	11.78	4.55	0.221	0.045
S3	L46	0.457	18.89	5.41	0.167	0.024
S3	L39	0.794	20.62	7.36	0.195	0.039
S3	L39	1.003	21.58	4.74	0.210	0.047
S3	L39	0.380	16.12	7.67	0.146	0.024
S3	L39	0.757	21.04	6.73	0.159	0.036
S3	L39	0.476	13.61	7.48	0.289	0.035
S4	L55	0.585	12.51	17.58	0.215	0.047
S4	L55	0.423	26.58	15.93	0.130	0.016

S4	L55	0.125	7.45	8.96	0.066	0.017
S4	L55	0.464	9.80	7.81	0.150	0.047
S4	L55	0.350	13.20	6.50	0.067	0.027
S4	L56	0.361	14.57	9.75	0.321	0.025
S4	L56	0.804	24.22	17.33	0.258	0.033
S4	L56	0.866	20.92	5.08	0.347	0.041
S4	L56	0.373	19.82	5.27	0.091	0.019
S4	L56	0.326	14.88	7.37	0.161	0.022
S4	L57	0.969	23.18	17.72	0.331	0.042
S4	L57	2.650	22.59	4.90	0.323	0.117
S4	L57	0.475	25.15	10.04	0.133	0.019
S4	L57	0.965	15.20	5.87	0.188	0.064
S4	L57	0.330	20.26	6.48	0.087	0.016
S4	L58	1.319	18.30	16.90	0.511	0.072
S4	L58	0.778	12.16	21.00	0.414	0.064
S4	L58	0.964	15.40	19.33	0.340	0.063
S4	L58	1.250	15.24	6.29	0.369	0.082
S4	L58	0.496	16.82	5.95	0.182	0.030
S4	L60	1.087	24.64	19.11	0.445	0.044
S4	L60	0.456	11.36	10.49	0.540	0.040
S4	L60	0.702	10.34	4.01	0.340	0.068
S4	L60	0.563	13.93	9.86	0.306	0.040
S4	L60	0.278	12.81	6.59	0.207	0.022
S4	L61	0.425	10.90	8.33	0.223	0.039
S4	L61	0.098	7.91	11.87	0.053	0.012
S4	L61	1.305	20.68	6.10	0.174	0.063
S4	L61	0.292	15.52	15.32	0.077	0.019
S4	L61	0.236	19.87	5.89	0.054	0.012
S4	L62	0.351	22.49	17.43	0.256	0.016
S4	L62	0.358	9.58	18.47	0.258	0.037
S4	L62	0.105	8.36	8.94	0.084	0.013
S4	L62	0.123	6.66	4.71	0.159	0.019
S4	L64	0.517	16.95	20.69	0.299	0.031
S4	L64	0.476	17.19	18.86	0.288	0.028
S4	L64	0.519	4.35	5.45	0.217	0.119
S4	L64	0.226	11.22	6.83	0.153	0.020
S4	L64	0.044	6.30	11.01	0.030	0.007
S4	L67	0.264	12.22	21.31	0.204	0.022
S4	L67	0.237	11.67	10.34	0.175	0.020
S4	L67	1.998	24.02	4.66	0.256	0.083
S4	L67	0.630	24.82	14.31	0.128	0.025
S4	L67	0.636	17.56	5.30	0.103	0.036
S4	L70	0.810	17.68	18.83	0.393	0.046
S4	L70	0.535	16.57	20.12	0.469	0.032
S4	L70	2.363	15.60	2.77	0.417	0.152
S4	L70	1.005	20.85	17.67	0.205	0.048

S4	L70	0.560	18.91	18.18	0.166	0.030
S4	L71	1.702	26.02	21.75	0.342	0.065
S4	L71	0.588	23.91	19.38	0.263	0.025
S4	L71	1.466	20.30	6.49	0.374	0.072
S4	L71	0.209	18.20	7.10	0.075	0.012
S4	L71	2.537	23.87	4.80	0.388	0.106
S4	L73	0.288	7.92	6.78	0.257	0.036
S4	L73	0.748	14.01	3.55	0.264	0.053
S4	L73	0.941	9.92	5.20	0.462	0.095
S4	L73	0.618	23.16	11.67	0.208	0.027
S4	L73	0.843	18.78	9.47	0.194	0.045
S5	L74	0.278	10.64	8.27	0.068	0.026
S5	L74	0.059	5.91	11.42	0.023	0.010
S5	L74	0.188	9.71	5.39	0.121	0.019
S5	L74	0.062	6.80	11.58	0.050	0.009
S5	L75	0.569	19.28	8.19	0.101	0.030
S5	L75	0.159	9.91	9.87	0.080	0.016
S5	L75	0.152	13.85	10.97	0.064	0.011
S5	L75	0.505	19.82	5.88	0.175	0.026
S5	L75	0.303	8.10	11.34	0.091	0.037
S5	L76	0.441	20.61	11.49	0.091	0.021
S5	L76	0.446	11.81	8.03	0.208	0.038
S5	L76	0.303	18.27	10.5	0.128	0.017
S5	L76	0.430	14.72	11.57	0.094	0.029
S5	L76	0.071	10.33	4.93	0.030	0.007
S5	L77	0.321	16.38	9.22	0.072	0.020
S5	L77	0.129	8.38	9.65	0.102	0.015
S5	L77	0.531	14.30	11.88	0.112	0.037
S5	L77	0.349	10.93	4.59	0.081	0.032
S5	L77	0.572	21.84	10.56	0.074	0.026
S5	L83	0.293	26.16	12.31	0.021	0.011
S5	L83	0.805	19.67	6.05	0.133	0.041
S5	L83	1.709	18.20	8.29	0.207	0.094
S5	L83	0.356	16.70	10.67	0.076	0.021
S5	L84	0.498	13.42	8.01	0.113	0.037
S5	L84	0.426	11.98	9.03	0.123	0.036
S5	L84	0.443	13.26	13.81	0.065	0.033
S5	L84	0.971	14.75	5.41	0.189	0.066
S5	L84	0.392	11.77	8.48	0.090	0.033
S5	L85	0.265	6.56	7.92	0.094	0.040
S5	L85	0.198	7.02	9.97	0.085	0.028
S5	L85	0.239	16.58	13.94	0.054	0.014
S5	L85	0.432	12.67	6.32	0.121	0.034
S5	L85	0.536	20.06	7.85	0.166	0.027
S5	L87	1.022	18.22	7.74	0.173	0.056
S5	L87	0.174	10.85	11.27	0.071	0.016

S5	L87	0.035	14.16	14.86	0.028	0.003
S5	L87	0.660	12.7	6.26	0.163	0.052
S5	L87	0.645	16.75	7.70	0.227	0.039
S5	L88	0.819	21.21	9.70	0.077	0.039
S5	L88	0.550	14.79	8.16	0.136	0.037
S5	L88	0.527	17.86	9.07	0.074	0.030
S5	L88	0.372	11.90	6.90	0.085	0.031
S5	L88	0.249	10.33	9.88	0.057	0.024
S5	L89	0.337	9.76	10.43	0.083	0.035
S5	L89	0.147	7.69	7.86	0.101	0.019
S5	L89	0.421	20.26	10.22	0.063	0.021
S5	L89	0.416	14.82	5.80	0.120	0.028
S5	L89	0.224	8.74	10.00	0.042	0.026
S5	L90	0.296	12.97	10.37	0.056	0.023
S5	L90	0.451	18.32	7.67	0.086	0.025
S5	L90	0.243	19.94	10.82	0.033	0.012
S5	L90	0.888	19.90	4.56	0.161	0.045
S5	L90	0.221	10.50	9.02	0.067	0.021
S5	L92	0.490	16.44	10.46	0.035	0.030
S5	L92	0.931	21.36	10.28	0.080	0.044
S5	L92	1.654	18.77	5.13	0.213	0.088
S5	L92	1.404	26.30	7.98	0.104	0.053
S5	L82	0.130	7.61	8.63	0.045	0.017
S5	L82	0.124	7.88	9.39	0.042	0.016
S5	L82	0.194	10.86	9.43	0.087	0.018
S5	L82	0.142	6.29	11.66	0.052	0.023
S5	L82	0.090	8.89	10.58	0.063	0.010
S5	L114	0.364	16.02	7.67	0.089	0.023
S5	L114	0.370	10.45	8.48	0.078	0.035
S5	L114	0.415	20.24	8.59	0.064	0.021
S5	L114	0.267	17.43	8.79	0.070	0.015
S5	L114	0.200	11.35	8.29	0.036	0.018
S6	L82	0.570	16.28	5.07	0.104	0.035
S6	L82	0.436	12.47	4.48	0.112	0.035
S6	L82	0.304	11.27	8.12	0.096	0.027
S6	L82	0.538	12.22	6.99	0.131	0.044
S6	L93	1.182	30.30	7.32	0.106	0.039
S6	L93	0.935	30.16	7.76	0.088	0.031
S6	L93	0.423	11.13	6.53	0.068	0.038
S6	L93	NA	NA	NA	NA	NA
S6	L93	2.408	17.97	4.7	0.204	0.134
S6	L94	0.485	16.71	6.75	0.075	0.029
S6	L94	0.629	23.31	5.31	0.050	0.027
S6	L94	0.601	19.40	5.61	0.139	0.031
S6	L94	0.265	16.54	6.37	0.040	0.016
S6	L94	0.100	11.08	7.15	0.015	0.009

S6	L95	2.725	15.31	4.00	0.389	0.178
S6	L95	2.569	16.36	6.20	0.195	0.157
S6	L95	4.237	12.28	3.19	0.476	0.345
S6	L95	1.068	19.07	5.52	0.075	0.056
S6	L95	5.570	21.18	4.15	0.343	0.263
S6	L96	1.832	24.11	4.65	0.181	0.076
S6	L96	2.300	31.50	6.01	0.240	0.073
S6	L96	1.671	19.21	5.12	0.150	0.087
S6	L96	1.336	11.93	4.39	0.258	0.112
S6	L96	2.198	25.56	5.81	0.184	0.086
S6	L97	0.580	15.68	6.68	0.063	0.037
S6	L97	1.316	34.64	6.56	0.038	0.038
S6	L97	1.395	53.67	5.16	0.058	0.026
S6	L97	0.328	18.21	8.42	0.029	0.018
S6	L98	1.015	11.53	6.30	0.184	0.088
S6	L98	1.777	34.84	5.36	0.089	0.051
S6	L98	2.991	25.56	3.79	0.214	0.117
S6	L98	1.925	26.73	6.17	0.132	0.072
S6	L99	2.648	29.42	4.70	0.250	0.090
S6	L99	2.201	34.93	4.88	0.233	0.063
S6	L99	5.024	34.18	3.75	0.282	0.147
S6	L99	3.721	31.53	3.32	0.301	0.118
S6	L99	3.260	27.17	5.77	0.232	0.120
S6	L100	0.236	29.56	6.75	0.016	0.008
S6	L100	1.478	23.09	4.17	0.193	0.064
S6	L100	1.175	24.47	4.96	0.095	0.048
S6	L100	0.754	22.85	6.42	0.098	0.033
S6	L102	0.917	20.84	6.76	0.126	0.044
S6	L102	2.674	23.46	6.25	0.166	0.114
S6	L102	2.188	16.33	5.6	0.178	0.134
S6	L102	2.397	26.34	5.27	0.179	0.091
S6	L102	0.995	13.45	6.96	0.181	0.074
S6	L104	0.616	16.66	6.00	0.099	0.037
S6	L104	2.238	13.9	4.25	0.291	0.161
S6	L104	3.378	34.47	5.46	0.138	0.098
S6	L104	4.163	34.98	6.12	0.107	0.119
S6	L105	2.651	28.81	4.32	0.146	0.092
S6	L105	1.447	27.3	4.64	0.156	0.053
S6	L105	2.212	22.12	5.63	0.159	0.100
S6	L105	1.322	25.43	6.86	0.073	0.052
S6	L105	0.609	22.57	6.17	0.080	0.027
S6	L106	1.074	14.51	3.82	0.372	0.074
S6	L106	2.407	22.08	5.86	0.172	0.109
S6	L106	4.680	31.41	5.55	0.243	0.149
S6	L106	1.162	20.03	6.11	0.237	0.058
S6	L114	0.963	24.08	5.04	0.089	0.040

S6	L114	0.576	17.99	6.39	0.088	0.032
S6	L114	0.880	26.67	5.88	0.099	0.033
S6	L114	0.474	22.55	5.31	0.061	0.021
S6	L114	1.362	25.69	3.99	0.089	0.053
S7	L107	1.056	10.56	9.88	0.036	0.100
S7	L107	0.593	19.77	11.11	0.120	0.030
S7	L107	0.571	27.18	12.53	0.113	0.021
S7	L107	0.774	22.76	11.3	0.148	0.034
S7	L107	1.832	33.30	8.64	0.152	0.055
S7	L108	1.425	22.98	9.45	0.268	0.062
S7	L108	1.405	15.11	12.57	0.188	0.093
S7	L108	2.873	41.64	9.21	0.245	0.069
S7	L109	1.639	17.07	7.55	0.540	0.096
S7	L109	4.342	22.85	11.83	0.458	0.190
S7	L109	0.874	24.28	13.92	0.130	0.036
S7	L109	0.841	16.81	11.74	0.297	0.050
S7	L109	1.576	25.83	8.91	0.196	0.061
S7	L110	0.346	17.28	11.24	0.060	0.020
S7	L112	0.318	22.73	14.43	0.061	0.014
S7	L112	0.467	15.08	9.66	0.113	0.031
S7	L112	1.017	32.81	7.86	0.142	0.031
S7	L112	2.100	24.14	7.78	0.278	0.087
S7	L112	1.909	26.15	8.98	0.131	0.073
S7	L113	0.312	22.26	12.77	0.056	0.014
S7	L113	0.502	12.56	12.58	0.254	0.040
S7	L113	0.521	14.09	6.32	0.255	0.037
S7	L113	1.748	27.75	7.85	0.303	0.063
S7	L113	0.588	18.97	8.31	0.170	0.031
S7	L116	0.876	21.89	12.43	0.127	0.040
S7	L116	2.388	20.95	13.31	0.429	0.114
S7	L116	1.660	27.21	7.16	0.208	0.061
S7	L116	2.560	26.67	8.26	0.343	0.096
S7	L116	1.691	26.42	9.85	0.084	0.064
S7	L117	0.363	15.77	11.77	0.087	0.023
S7	L117	1.267	17.84	11.67	0.216	0.071
S7	L117	0.689	11.30	6.42	0.176	0.061
S7	L117	1.101	36.71	7.27	0.120	0.030
S7	L117	1.881	32.43	8.16	0.191	0.058
S7	L118	0.185	14.23	16.09	0.064	0.013
S7	L118	1.563	36.35	11.63	0.119	0.043
S7	L118	1.543	28.06	8.53	0.280	0.055
S7	L118	2.482	42.06	7.82	0.234	0.059
S7	L118	4.283	57.11	9.04	0.199	0.075
S7	L119	0.915	24.73	14.52	0.156	0.037
S7	L119	1.479	25.06	10.38	0.242	0.059
S7	L119	0.283	14.87	8.72	0.075	0.019

S7	L119	2.019	36.06	8.04	0.342	0.056
S7	L119	0.610	17.93	9.53	0.083	0.034
S7	L120	0.308	13.37	13.94	0.082	0.023
S7	L120	0.267	11.62	10.32	0.105	0.023
S7	L120	0.211	9.60	8.32	0.117	0.022
S7	L120	0.203	12.68	9.59	0.074	0.016
S7	L120	0.507	19.49	8.54	0.049	0.026
S7	L121	1.375	28.65	12.79	0.220	0.048
S7	L121	1.953	18.96	14.52	0.300	0.103
S7	L121	2.381	27.06	6.55	0.513	0.088
S7	L121	5.204	56.57	8.01	0.512	0.092
S7	L121	6.011	28.76	6.83	0.330	0.209
S7	L123	1.215	35.74	15.43	0.215	0.034
S7	L123	1.266	39.56	18.29	0.206	0.032
S7	L123	2.169	32.87	16.83	0.250	0.066
S7	L123	1.107	30.74	16.53	0.212	0.036
S7	L123	0.927	25.06	15.56	0.193	0.037
S7	L125	0.519	13.31	14.69	0.172	0.039
S7	L125	0.738	21.09	14.00	0.256	0.035
S7	L125	0.580	24.18	15.41	0.152	0.024
S7	L125	1.206	22.34	15.97	0.290	0.054
S7	L125	1.059	27.16	14.09	0.271	0.039

Table S2: Relative abundances of the 15 main bacterial phyla in the OTUs identified from NGS sequencing of DNA extracts from the rhizospheric soil of 9 pearl millet inbred lines, as well as from control unplanted soil (T). Kruskal-Wallis and Dunn Pairwise tests were performed to compare rank of phylum relative abundance between lines and control unplanted soil (p = 0.05). Different letters indicate significant differences between samples in the same row (Kruskal-Wallis test + Dunn Pairwise comparison at p < 0.05). (* vs **) indicates significant differences between the two groups of inbred lines (Kruskal-Wallis test p <0.05). *sd* indicates standard deviations.

	Low RAS/RT			High RAS/RT								Low RAS/RT	High RAS/RT
Phylum	L8	L82	L44	L92	L12	L118	L14	L57	L39	T			
**Proteobacteria**	38.4	29.5	33.8	36.4	36.3	**39.1**	**39.6**	36.6	**42.6**	36.9	Mean	*** 33.9**	**** 38.4**
	bcd	a	ab	abc	abc	cd	cd	abc	d	abcd	*sd*	*4.4*	*2.5*
**Firmicutes**	21.1	**36.5**	**25**	16.1	18.6	17.3	18.2	20.1	18.5	18.9		**** 27.5**	*** 18.1**
	bc	c	bc	a	ab	ab	ab	abc	ab		*sd*	*8*	*1.3*
**Actinobacteria**	15.9	14.4	22.5	23.4	20.1	22.5	19.1	18.2	15.6	20.9		**17.6**	**19.8**
	ab	a	d	d	d	d	bcd	abcd	abc	cd	*sd*	*4.3*	*2.9*
**Bacteroidetes**	6.6	4.3	6.1	5.2	5.2	6.2	6.5	5.5	5.7	4.3		**5.7**	**5.7**
	c	a	bc	ab	abc	bc	c	abc	abc	a	*sd*	*1.2*	*0.6*
**Acidobacteria**	5.6	4.5	3.5	5.7	6.2	4.3	4.2	5.8	4.6	5.5		**4.5**	**5.1**
	cd	abc	a	cd	d	abc	ab	cd	abc	bcd	*sd*	*1.1*	*0.9*
**Chloroflexi**	5.1	4.6	4	6.2	6.1	4.5	5.6	6.3	5.4	6.2		**4.6**	**5.7**
	abcd	abc	a	cde	de	ab	bcde	cde	adcde	e	*sd*	*0.5*	*0.7*
**Planctomycetes**	2.9	2.6	2.3	3.2	3.3	2.7	2.8	3.5	3.4	3.1		**2.6**	**3.2**
	abcd	ab	a	bcd	bcd	ab	abc	cd	d	bcd	*sd*	*0.3*	*0.3*
**Gemmatimonadetes**	1.8	1.5	1.3	1.7	2.1	1.5	1.8	2	1.7	2.2		**1.5**	**1.8**

	abcde	abc	a	abcde	de	ab	bcde	cde	adcd	e	*sd*	*0.3*	*0.2*
**Verrucomicrobia**	1.4	1	0.8	1	1	1	1.2	1	1.3	0.8		**1.1**	**1.1**
	d	bcd	ab	abc	abcd	abc	cd	abc	cd	a	*sd*	*0.3*	*0.1*
**Nitrospirae**	0.6	0.5	0.4	0.6	0.6	0.5	0.6	0.7	0.6	0.7		**0.5**	**0.6**
	cd	abc	a	abc	abc	ab	abc	cd	bcd	d	*sd*	*0.1*	*0.1*
**Armatimonadetes**	0.3	0.3	0.2	0.3	0.3	0.2	0.2	0.3	0.3	0.3		**0.25**	**0.3**
	cd	abcd	a	bcd	d	abc	ab	abcd	abcd	cd	*sd*	*0.05*	*0.1*
**Fibrobacteres**	0.07	0.06	0.04	0.1	0.09	0.07	0.16	0.07	0.1	0.04		**0.05**	**0.1**
	abc	abc	ab	bcd	cd	bcd	d	abcd	cd	a	*sd*	*0.01*	*0.03*
**Parcubacteria**	0.05	0.04	0.05	0.03	0.04	0.04	0.08	0.04	0.07	0.06		**0.05**	**0.05**
	bcd	ab	abc	a	ab	abc	d	abc	cd	bcd	*sd*	*0.01*	*0.02*
**Chlamydiae**	0.05	0.04	0.03	0.03	0.03	0.04	0.05	0.02	0.05	0.05		**0.04**	**0.04**
	bc	abc	abc	ab	abc	abc	abc	a	c	c	*sd*	*0.01*	*0.01*
**Elusimicrobia**	0.04	0.04	0.03	0.03	0.04	0.03	0.03	0.04	0.04	0.06		**0.03**	**0.04**
	ab	ab	a	a	ab	a	a	ab	ab	b	*sd*	*0.01*	*0.01*
**Sequences count**	**207209**	**282184**	**263447**	**223039**	**243546**	**265338**	**225013**	**238563**	**235656**	***222501***			

Tableau S3: Relative distribution of the 26 main bacterial orders in the rhizosphere (root-adhering soil fraction) and in the control soil (T). Different letters indicate significant differences between samples in the same row (Kruskal-Wallis test + Dunn Pairwise comparison at $p < 0.05$). (* vs **) indicates significant differences between the two groups of inbred lines (Kruskal-Wallis test $p <0.05$).

	**Low RAS/RT ines**			**High RAS/RT lines**								
Orders	L8	L82	L44	L118	L92	L12	L57	L39	L14	(T)	**Low RAS/RT**	**High RAS/RT**
o__Bacillales	34.15	50.86	34.20	25.42	24.04	29.14	30.71	29.29	28.79	30.09	****39.74**	***27.90**
	bc	c	ab	a	a	ab	abc	ab	ab	abc		
o__Rhizobiales	20.03	14.71	17.27	20.26	20.71	21.96	22.42	24.69	23.14	24.04	***17.34**	****22.19**
	abc	a	ab	abcd	abcd	bcde	cde	e	cde	de		
o__Actinomycetales	9.80	8.84	19.31	20.39	19.42	13.24	10.61	7.93	10.68	9.68	**12.65**	**13.71**
	ab	a	c	c	c	bc	abc	a	abc	ab		
o__Sphingomonadales	6.14	4.31	6.08	7.24	7.53	6.42	5.75	6.52	7.45	5.35	**5.51**	**6.82**
	ab	a	ab	b	b	ab	ab	ab	b	ab		
o__Rubrobacterales	3.99	3.36	2.95	3.19	3.35	3.66	3.83	3.44	3.55	4.31	**3.43**	**3.50**
	ab	a	a	a	a	ab	ab	ab	ab	b		
o__Burkholderiales	3.89	3.05	2.70	3.81	2.48	3.17	3.14	4.92	2.98	3.26	**3.21**	**3.42**
	bc	ab	a	a	a	abc	abc	c	abc	abc		
o__Saprospirales	2.74	1.48	1.62	2.41	1.71	2.31	2.26	2.23	3.08	1.86	**1.95**	**2.33**
	d	a	ab	bcd	abc	cd	bcd	bcd	d	abc		
o__Xanthomonadales	2.34	1.29	2.18	2.46	2.35	2.11	2.27	3.02	2.58	1.95	**1.94**	**2.46**
	bc	a	bc	bc	abc	abc	bc	c	bc	ab		
o__RB41	1.39	1.04	0.63	0.85	1.58	1.76	1.36	0.97	0.90	1.23	**1.02**	**1.24**
	de	abcd	a	ab	cde	e	bcde	abc	abc	bcde		
o__Solirubrobacterales	1.24	1.03	1.27	1.34	1.49	1.89	1.74	1.85	2.25	3.06	**1.18**	**1.76**
	ab	a	abc	abc	abcd	cde	bcde	cde	de	e		
o__Cytophagales	0.72	0.43	1.86	1.08	0.76	0.50	0.67	0.66	1.07	0.33	**1.00**	**0.79**
	bc	ab	c	c	bc	ab	abc	abc	c	a		
c__Acidobacteria-6;o__iii1-15	0.87	0.73	0.67	0.79	1.09	1.14	1.27	0.75	0.82	0.98	**0.75**	**0.98**

	abc	ab	a	ab	abc	bc	c	a	abc	abc		
c__Gemm-1;o__	0.85	0.71	0.75	0.77	0.93	0.95	1.05	0.78	0.97	1.11	**0.77**	**0.91**
	abc	a	ab	a	abcd	abcd	cd	a	bcd	d		
o__Rhodospirillales	0.61	0.49	0.39	0.52	0.56	0.73	0.64	0.56	0.53	0.66	**0.50**	**0.59**
	bcde	ab	a	abcd	abcd	e	cde	abcd	abc	de		
o__Myxococcales	1.50	1.24	0.56	1.02	0.88	1.04	1.49	2.04	1.81	1.24	**1.10**	**1.38**
	bc	abc	a	ab	ab	ab	bc	bc	c	abc		
o__Micrococcales	0.32	0.26	0.23	0.24	0.34	0.30	0.45	0.34	0.25	0.27	**0.27**	**0.32**
	ab	ab	a	a	ab	ab	b	ab	ab	ab		
c__Ellin6529;o__	0.48	0.44	0.42	0.52	0.67	0.81	0.74	0.71	0.84	1.33	**0.45**	**0.72**
	ab	ab	a	abc	abcd	cd	bcd	abcd	d	d		
o__[Roseiflexales]	0.37	0.28	0.22	0.26	0.45	0.40	0.48	0.38	0.37	0.37	**0.29**	**0.39**
	bcd	abc	a	ab	d	cd	cd	bcd	bcd	bcd		
o__Alteromonadales	0.81	0.18	0.30	0.11	0.24	0.10	0.29	0.32	0.37	0.07	**0.43**	**0.24**
	d	ab	abcd	abc	abcd	abc	bcd	cd	abcd	a		
o__Nitrospirales	0.37	0.26	0.22	0.23	0.26	0.28	0.35	0.35	0.29	0.41	**0.28**	**0.29**
	bc	ab	a	a	ab	ab	bc	bc	abc	c		
o__Caulobacterales	0.29	0.15	0.18	0.23	0.27	0.27	0.23	0.33	0.41	0.38	**0.21**	**0.29**
	abc	a	a	ab	bc	bc	ab	bc	bc	c		
o__Gaiellales	0.20	0.19	0.21	0.23	0.22	0.26	0.27	0.24	0.29	0.34	**0.20**	**0.25**
	ab	a	abc	abc	abcd	abcd	bcd	abcd	cd	d		
c__Thermomicrobia; o__JG30-KF-CM45	0.31	0.19	0.22	0.18	0.41	0.41	0.37	0.30	0.35	0.46	**0.24**	**0.34**
	abcd	ab	abc	a	cd	d	bcd	abcd	cd	d		
o__Rhodobacterales	0.33	0.21	0.75	0.89	0.62	0.42	0.18	0.23	0.23	0.13	**0.43**	**0.43**
	bc	ab	c	c	c	bc	ab	ab	ab	a		
o__Pseudomonadales	0.31	0.10	0.06	1.07	0.24	0.16	0.01	0.51	0.03	0.02	**0.15**	**0.34**
	d	cd	abcd	abcd	abcd	abcd	a	bcd	abc	abc		
o__Nitrososphaerales	5.93	4.19	4.74	4.51	7.40	6.56	7.41	6.65	5.97	7.08	**4.95**	**6.42**
	abc	a	ab	ab	c	bc	c	bc	abc	c		

Table S4: Taxonomic distribution of 184 bacterial EPS-producing strains isolated on C-enriched TSA/10 media from the rhizosphere (root adhering soil fraction) of 9 pearl millet inbred lines.

	Low RAS/RT			High RAS/RT						
	L8	L82	L44	L92	L12	L118	L14	L57	L39	Total
Agrobacterium fabrum	0	0	0	0	0	0	2	0	0	2
Arthrobacter chlorophenolicus	0	0	0	0	2	0	0	0	1	3
Arthrobacter crystallopoietes	0	0	0	0	1	0	0	0	0	1
Arthrobacter defluvii	0	0	0	0	6	0	0	1	0	7
Arthrobacter globiformis	0	1	2	1	0	1	4	1	3	13
Arthrobacter oryzae	0	0	0	0	1	0	0	0	0	1
Arthrobacter pascens	0	2	3	1	13	1	3	0	5	28
Arthrobacter phenanthrenivorans Sphe3	0	0	0	0	0	0	0	0	1	1
Arthrobacter tecti	0	0	0	0	0	0	0	1	0	1
Arthrobacter ureafaciens	0	0	0	0	1	0	0	0	0	1
Bacillus aryabhattai	0	3	1	2	9	0	2	3	2	22
Bacillus beveridgei	0	0	0	0	0	0	0	0	1	1
Bacillus isabeliae	0	0	1	0	0	0	0	0	0	1
Bacillus megaterium	1	0	0	0	0	0	0	0	0	1
Bacillus megaterium NBRC 15308 = ATCC 14581	0	0	0	2	0	0	0	0	0	2
Bacillus pervagus	0	0	0	0	1	0	0	0	0	1
Bacillus tequilensis	0	0	1	0	0	0	0	0	0	1
Corynebacterium ilicis	0	0	0	0	1	0	0	0	0	1
Cupriavidus taiwanensis LMG 19424	0	2	0	0	0	0	0	0	0	2
Enterobacter hormaechei	0	0	0	0	0	0	1	0	0	1
Enterobacter xiangfangensis	0	1	0	0	0	0	2	0	0	3
Exiguobacterium indicum	0	2	0	0	0	0	0	0	0	2
Franconibacter helveticus	0	0	0	0	1	0	0	0	0	1

Glycomyces halotolerans	0	0	0	0	0	1	0	0	0	1
Halolactibacillus alkaliphilus	0	1	0	0	0	0	0	0	0	1
Microbacterium barkeri	2	0	9	1	3	4	3	2	6	30
Microbacterium paraoxydans	0	0	0	0	1	0	0	0	0	1
Ochrobactrum pseudogrignonense	0	0	0	0	0	0	0	0	5	5
Paenibacillus mucilaginosus KNP414	0	1	0	0	0	0	0	0	0	1
Paenibacillus peoriae KCTC 3763	0	0	0	0	0	0	1	0	0	1
Paenibacillus polymyxa	0	0	1	1	1	0	2	1	0	6
Paenibacillus polymyxa E681	0	0	1	0	0	0	0	0	0	1
Paenibacillus polymyxa SC2	1	0	0	3	0	0	4	1	0	9
Paracoccus halophilus	0	0	0	0	0	0	1	0	0	1
Pseudomonas benzenivorans	0	0	0	0	0	0	1	0	0	1
Pseudomonas monteilii	1	0	0	1	0	0	0	0	1	3
Pseudomonas plecoglossicida	0	0	3	4	0	0	5	0	1	13
Pseudomonas putida	1	0	0	0	0	0	0	0	0	1
Rhizobium pusense	0	0	0	0	0	0	1	1	1	3
Rothia dentocariosa	0	0	0	0	0	0	0	0	1	1
Salmonella enterica	0	0	0	0	0	0	1	0	0	1
Sphingobacterium cladoniae	0	0	0	0	0	1	0	0	0	1
Staphilococcus auricularis	0	0	1	0	0	0	0	0	0	1
Stenotrophomonas pavanii	0	0	0	0	0	0	1	0	0	1
Streptomyces libani	0	0	1	0	0	0	0	0	0	1
Streptomyces pactum	0	0	0	0	0	0	1	0	0	1
Sulfirihydrogenibium krisjanssonii	0	0	0	0	1	0	0	0	0	1
Variovorax paradoxus S110	0	0	0	0	1	0	0	0	0	1
Total	6	13	24	16	43	8	35	11	28	184
